# Differential DNA methylation in high-grade serous ovarian cancer (HGSOC) is associated with tumor behavior

**DOI:** 10.1038/s41598-019-54401-w

**Published:** 2019-11-29

**Authors:** Henry D. Reyes, Eric J. Devor, Akshaya Warrier, Andreea M. Newtson, Jordan Mattson, Vincent Wagner, Gabrielle N. Duncan, Kimberly K. Leslie, Jesus Gonzalez-Bosquet

**Affiliations:** 1 Department of Obstetrics and Gynecology, University of Iowa Carver College of Medicine, Iowa City, IA 52242 USA; 2 Holden Comprehensive Cancer Center, University of Iowa Hospitals and Clinics, Iowa City, IA 52242 USA; 3 Present Address: Department of Obstetrics and Gynecology, Division of Gynecologic Oncology, University at Buffalo, Buffalo, NY USA

**Keywords:** Ovarian cancer, Prognostic markers

## Abstract

The epigenome offers an additional facet of cancer that can help categorize patients into those at risk of disease, recurrence, or treatment failure. We conducted a retrospective, nested, case-control study of advanced and recurrent high-grade serous ovarian cancer (HGSOC) patients in which we assessed epigenome-wide association using Illumina methylationEPIC arrays to characterize DNA methylation status and RNAseq to evaluate gene expression. Comparing HGSOC tumors with normal fallopian tube tissues we observe global hypomethylation but with skewing towards hypermethylation when interrogating gene promoters. In total, 5,852 gene interrogating probes revealed significantly different methylation. Within HGSOC, 57 probes highlighting 17 genes displayed significant differential DNA methylation between primary and recurrent disease. Between optimal vs suboptimal surgical outcomes 99 probes displayed significantly different methylation but only 29 genes showed an inverse correlation between methylation status and gene expression. Overall, differentially methylated genes point to several pathways including RAS as well as hippo signaling in normal vs primary HGSOC; valine, leucine, and isoleucine degradation and endocytosis in primary vs recurrent HGSOC; and pathways containing immune driver genes in optimal vs suboptimal surgical outcomes. Thus, differential DNA methylation identified numerous genes that could serve as potential biomarkers and/or therapeutic targets in HGSOC.

## Introduction

Ovarian cancer is the fifth leading cause of death in women and is responsible for more deaths than any other gynecologic malignancy, with an estimated 22,530 new cases and 13,980 related deaths in the US for 2019^1,2^. Unfortunately, screening modalities and multimodal screening algorithms are inadequate for predicting patients who will develop ovarian cancer^3–7^. Tubo-ovarian high-grade serous carcinomas account for about 70% of ovarian carcinomas and some 85% of new cases are diagnosed at an advanced stage. For these advanced cases (Stages III–IV), the five year relative survival rate is poor at around 30–40% compared to early-stage cancer confined to the pelvis (Stages I–II) at 70%^8,9^.

Recommended treatment for advanced ovarian cancer consists of primary cytoreductive/debulking surgery followed by 6 to 8 cycles of combination chemotherapy with a platinum agent and taxane per National Comprehensive Cancer Network (NCCN) guidelines unless there are contraindications for the initial surgical approach, in which case neoadjuvant treatment followed by possible interval debulking surgery and additional cycles of chemotherapy can be considered^10,11^. The goal of surgery is for optimal (<1 cm residual disease) or complete cytoreduction (no visible residual disease) in order to achieve the highest possible survival benefit^12^. Unfortunately, not everyone will be optimally debulked and the majority of patients diagnosed at an advanced stage will most likely develop recurrence (80%) after initial treatment response with a median interval of 16 months^13,14^.

The goal of 21^st^ century precision medicine is to identify clinically relevant groups of patients based upon shared clinicopathological and molecular characteristics in order to deliver the most efficacious treatment. Next-generation molecular technologies assess the entire genome, and the molecular characterization of ovarian cancers conducted by the Cancer Genome Atlas research network (TCGA) was a major step toward this effort. TP53 mutations were found in 96% of high-grade serous ovarian cancers (HGSOCs), while dysregulation in the NOTCH, RB1, and PI3K/RAS signaling pathways were detected in 22%, 67%, and 45%, respectively. Alterations in genes involved in the homologous recombination repair pathway (BRCA1 and BRCA2, EMSY, and Fanconi anemia genes) were noted in 51% of cases, while overexpression of CCNE1 and FOXM1 were associated with lower overall survival^15^. Although the TCGA identified important genetic alterations in HGSOC, the findings remain limited in terms of guiding treatment recommendations and predicting clinical outcomes. Genomic alterations are not the sole contributor to tumorigenesis, and the more we understand the complexities that govern ovarian cancer, the better equipped we will be to effectively treat patients^16^.

In addition to gene-specific alterations, epigenetic mechanisms contribute to differential gene regulation via histone tail modification and DNA methylation. These changes do not alter the sequence of the DNA strand but affect how tightly packed the DNA is and its ability to be accessed by transcription factors^17,18^. Methylation status of DNA has long been associated with carcinogenesis^19^. DNA methylation involves transfer of a methyl group to a cytosine base via DNA methyltransferase (DNMT). The resulting 5-methylcytosine then deaminates to thymine, creating a base change that leads to transcriptional repression. DNA methylation also induces recruitment of methyl binding proteins and histone deacetylases (HDAC) that results in a condensed, transcriptionally inaccessible chromatin structure^20^. This methylation process occurs exclusively in CpG dinucleotide sequences in the 5′ to 3′ direction primarily in clusters called CpG islands or in areas in close proximity to gene promoters^21–23^.

The objectives of our study are: (1) to assess differential DNA methylation globally between high grade serous ovarian tumors and normal fallopian tube controls; (2) to examine methylation status differences within HGSOC relative to important clinical outcomes such as recurrence and debulking status; and (3) to correlate methylation status with RNA expression. These data will further characterize epigenomic phenomena that contribute to heterogeneity in the biology and behavior of ovarian cancer and inform future therapeutic decision making.

## Materials and Methods

### Tissue procurement and processing

**Figure 1:**
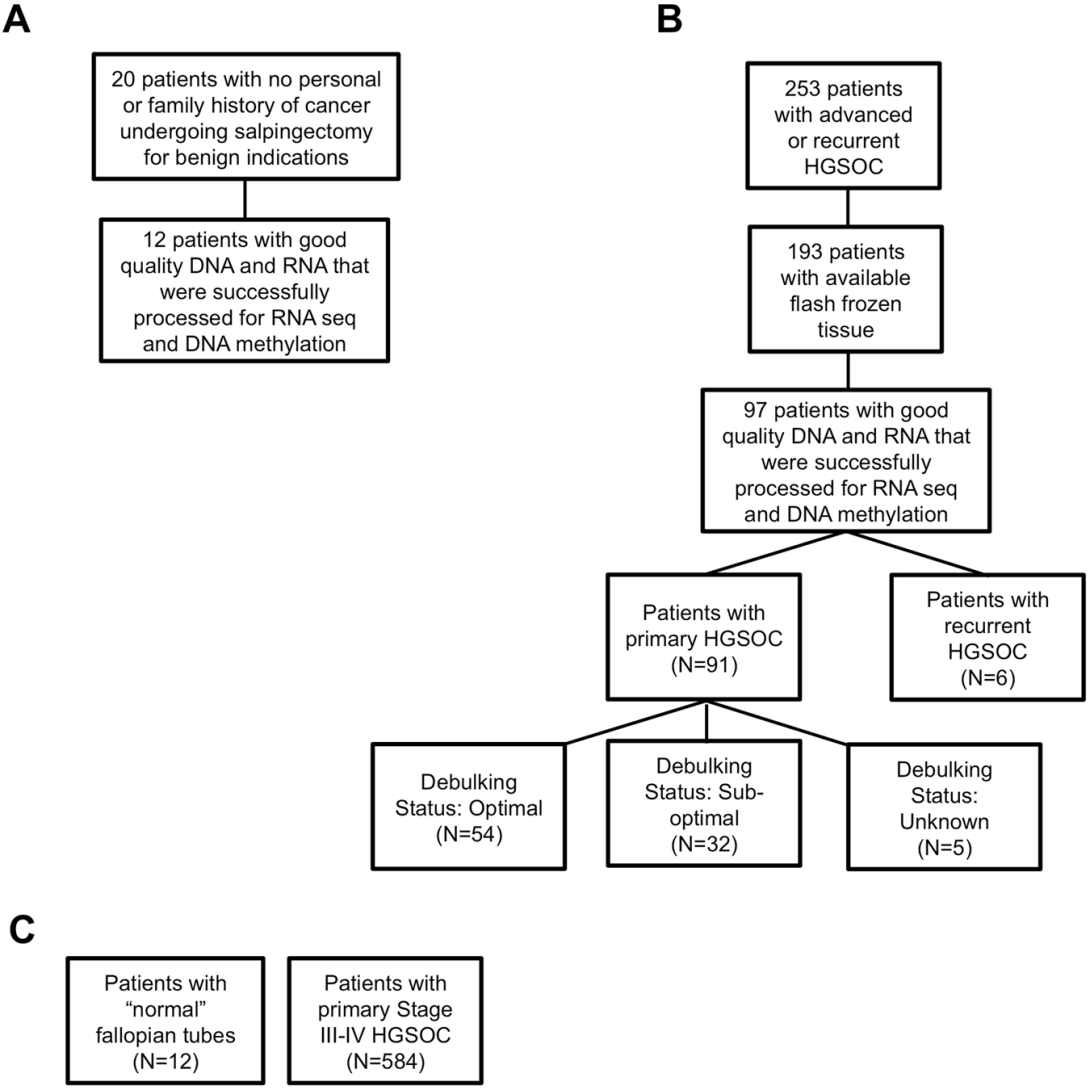
Distribution of controls and samples. (**A)** Normal fallopian tube controls retrieved from patients at the University of Iowa. (**B**) University of Iowa Hospitals and Clinics patients diagnosed with high grade serous ovarian cancer (HGSOC). (**C**) Normal fallopian tubes and HGSOC samples in the cancer genome atlas, TCGA, database.

We carried out a retrospective chart review to identify patients with advanced or recurrent HGSOC treated at the University of Iowa Hospitals and Clinics. We identified 253 such patients and determined that there were 193 with available flash-frozen tumor tissues stored in the Department of Obstetrics and Gynecology Gynecologic Oncology Bank (IRB, ID#200209010) that is part of the Women’s Health Tissue Repository (WHTR, IRB, ID#200910784)^24^. All tissues archived in the Gynecologic Oncology Bank were originally obtained from adult patients under informed consent in accordance with University of Iowa IRB guidelines. Both genomic DNA (gDNA) and total cellular RNA were purified from the identified tumors (Fig. 1).

A separate approval was given by the University of Iowa Institutional Review Board (IRB, ID#201202714) to collect 20 normal fallopian tube samples to be used as controls in coordination with the University of Iowa Tissue Procurement Core Facility. Again, all tissues were obtained from adult patients under informed consent in accordance with University of Iowa IRB guidelines. Samples came from the junction of the ampullary and fimbriated end of fallopian tubes of patients who were scheduled to undergo salpingectomy for benign indications. No patient indicating a personal or family history of cancer was included.

Genomic DNAs (gDNAs) were purified from frozen tumor tissues using the DNeasy Blood and Tissue Kit according to manufacturer’s (QIAGEN) recommendations. Yield and purity were assessed on a NanoDrop Model 2000 spectrophotometer and used a 260 nm/280 nm absorbance ratio of ~1.8 with minimal to no degradation as shown through horizontal agarose gel electrophoresis. Among the initial purifications, 97 gDNAs met our quality control standard which included minimal visible degradation (Fig. 1B). These samples were then bisulfite-converted using the EZ-96 Deep-Well Format DNA Methylation Kit (ZYMO Research) following the Illumina Infinium® Methylation Assay alternate incubation instructions. Similarly, gDNAs from the control fallopian tube tissues were purified and subjected to the quality control standard. Twelve of the original twenty tissues yielded material meeting our standard (Fig. 1A).

### Methylation assay

Bisulfite converted gDNAs from HGSOC tumors and fallopian tube controls were submitted to the Genomics Core Facility of the University of Iowa Institute of Human Genetics for processing on methylationEPIC arrays. The Illumina Infinium MethylationEPIC BeadChip Kit (Illumina, San Diego, CA, USA) allows quantification of more than 850,000 methylation sites across the human genome. Bisulfite-converted samples are denatured and neutralized before they are isothermally amplified overnight. The amplified product is fragmented enzymatically. After isopropanol precipitation, fragmented DNA is resuspended and placed onto Illumina methylationEPIC BeadChips and hybridized. There are two different bead types for each CpG locus, representing methylated or unmethylated DNA. The BeadChip is washed to remove unhybridized DNA, and the chip undergoes extension and staining. The arrays are scanned with the Illumina iScan, and methylation intensity is measured. Analysis is done using the Minfi R statistical package^25^.

A total of 97 samples, which included 91 primary and 6 recurrent patients, yielded usable data. Of the 91 patients who underwent primary debulking surgery, 86 had clinical information regarding their surgical cytoreduction outcomes; 54 were classified as “optimal” and 32 were classified as “sub-optimal” (Fig. 1B). Pre-operative clinical characteristics for the 97 patients whose tumors produced methylation and RNA sequencing data reported on here are shown in Table 1.

**Table 1:** Patient characteristics.

Pre-operative Characteristics	Primary HGSOC (n = 91)	Optimal Surgical Outcomes (n = 54)	Suboptimal Surgical Outcomes (n = 32)	p-value (Optimal vs suboptimal)
Age (mean)	60	62	58	0.239
BMI (mean)	26.7	25.9	27.8	0.227
Charlson Comorbidity Index	5.2	5.4	5.2	0.604
Preop CA-125	2,635	2,601	3,030	0.751
Disease in Upper abdomen (Other than Omentum) by Imaging	57	34	20	0.966
• Large bowel	3	1	1	0.708
• Spleen	0	0	0	N/A
• Mesenteric lymph node	4	2	1	0.888
• Porta/Hepatis	4	2	1	0.888
• Ascites (upper abdomen)	28	20	8	0.252
Other	24	12	11	0.224
• Disease in the Chest by Imaging	6	1	4	0.073
• Tumor	4	1	2	0.311
• Pleural effusion	4	1	2	0.311
• Neoadjuvant chemotherapy	14	10	4	0.496

All data refer to patients successfully sequenced.

### RNA sequencing

Total RNA was extracted from normal control and tumor tissues using the mirVana RNA purification kit (Thermo Fisher, Waltham, MA, USA) following manufacturer’s instructions. We initially evaluated RNA quality with NanoDrop Model 2000 spectrophotometer and used a 260 nm/280 nm absorbance ratio of ~2.0. RNAs with an RNA integrity number (RIN)^26^ of ≥7.0 via Trinean DropSense 16 spectrophotometer and Agilent Model 2100 bioanalyzer were then used for RNA sequencing. Messenger RNA-focused sequencing libraries were constructed using 500 ng of total RNA per sample that was converted to cDNA and linked to specific adaptors for sequencing via the Illumina TruSeq RNA library prep kit (Illiumina, San Diego, CA, USA). Indexed libraries for sequencing were re-concentrated into equimolar aliquots using the Agilent Model 2100 bioanalyzer and validated via an Illumina Library Quantification Kit (KAPA Biosystems, Wilmington, MA, USA). The Illumina HiSeq. 4000 genome sequencing platform employing 150 bp paired-end sequencing by synthesis chemistry was used. All library preparation and sequencing procedures were performed in the Genome Core Facility of the University of Iowa Institute of Human Genetics^27^.

### TCGA dataset validation

The TCGA methylation dataset contained 584 ovarian tumor samples and 12 normal fallopian tubes (Fig. 1C). Methylation was assessed via the Illumina Infinium HumanMethylation27 BeadChip array, which interrogates 27,578 CpG sites along the transcription start sites of 14,475 genes. We identified probes sharing similar nomenclature in the HumanMethylation27 BeadChip (used by TCGA) and the methylationEPIC (used in this study). Data from these probes were used to evaluate differential methylation between normal tissue and HGSOC in the TCGA dataset. The same statistical methods used in the Univeristy of Iowa Hospitals and Clinics (UIHC) data analysis were employed for the TCGA data. Evaluation of degree of agreement in the results of the TCGA and UIHC data was then performed using a receiver operating characteristic curve and C-statistics.

### Statistical analysis

Data from the methylation arrays were imported and processed using the Minfi R package. Results were reported as DNA methylation ratios (proportion of methylated/unmethylated), referred to as Beta. Beta values were then normalized using quantile normalization and subsequently log^2^-transformed to produce M values that were used for analysis as described^28^. Power calculations were carried out on all comparisons. Power at 50^th^ percentile of variance distribution estimated from log^2^-transformed gene expression data was determined to be at 90% with a sample size of at least 11 per group. A standard, two-tailed t- test was then performed to compare M values from 1) normal fallopian tube vs primary HGSOC, 2) primary HGSOC vs recurrent HGSOC, and 3) optimal vs suboptimal surgical outcomes. BRB-Array tools (http://linus.nci.nih.gov/BRB-ArrayTools.html) and R statistical packages were used for the analysis. When comparing differential methylation between normal fallopian tube controls vs primary ovarian cancer, a p-value of <10^−7^ and >2 fold change difference was considered statistically significant. When comparing differential methylation between primary vs recurrent ovarian cancer, and optimally vs suboptimally debulked cases, a p-value of < 10^−4^ was considered statistically significant.

Spearman’s rank correlation was used to analyze correlation between methylation and gene expression. A p-value of <0.05 was considered statistically significant.

Differential methylation of CpG dinucleotides clustered into regions was performed using 444,689 genomic region probes in the methylationEPIC chip. Minfi and Bumphunter bioconductor package were used to identify and analyze these segments. Longer segments (≥250 kilobase or Kb) of the DNA were probed for isolated loci of methylated CpGs that can have a gap between them of up to 0.5 Kb in the open sea regions, defined as >4 Kb away from the nearest CpG island. These loci were then grouped into clusters of ≤1.5 Kb and then averaged to produce a mean value for the cluster. This method of identifying long methylated DNA segments has been termed “block finding” and was made to identify long-range DNA alterations^28^. Shorter genomic regions of contiguous CpGs (CpG islands) found in the promoter regions that are <1 Kb in length were also identified and analyzed (also known as “bump hunting”) and is believed to be important in regulation of involved genes and thus cellular phenotype^28,29^. Analysis of variance was performed accounting for multiple comparisons. Statistical significance was met if p-value < 10^−3^ for long-range DNA methylation alterations and a p-value < 10^−4^ for short clusters of methylated, contiguous CpG regions. Different p-values were selected to better account for multiple comparisons, as in the Bonferroni method.

### Pathway analysis

Pathway enrichment was analyzed using clusterProfiler as described^30^ for classification and functional enrichment analysis of clusters. Individual genes represented by probes interrogating CpG sites of known genes differentially methylated between groups were then introduced to a wiring integration diagram database using the Kyoto Encyclopedia of Genes and Genomes (KEGG) (https://www.genome.jp/kegg/kegg1.html). “Known genes” refers to all of the ~25,000 genes that have been labeled by Illumina.

## Results

### Differential DNA methylation between normal fallopian tubes and primary HGSOC

**Figure 2:**
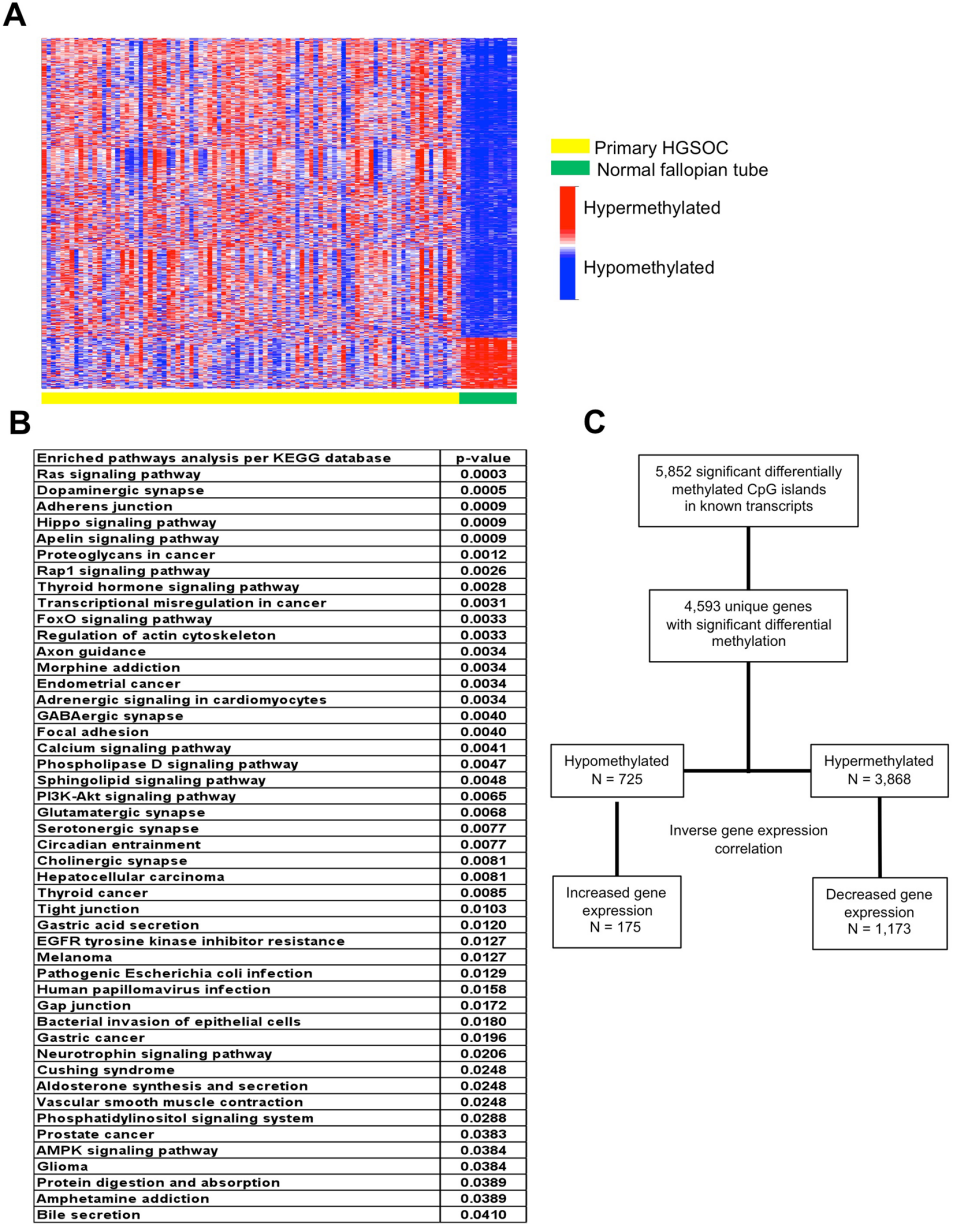
Differential methylation between normal fallopian tube controls and primary high-grade serous ovarian cancer. (**A**) Heat map clustering of methylation probes interrogating known genes for primary HGSOC and normal fallopian tubes in UIHC cohort. Each column represents a patient sample and each row represents a probe interrogating CpG sites of known genes categorized by Illumina. (**B**) Kyoto Encyclopedia of Genes and Genomes (KEGG) Pathways significantly enriched by genes that are differentially methylated between primary HGSOC and normal fallopian tube controls. (**C)** Experimental design showing how differentially methylated probes between HGSOC and fallopian tube controls were evaluated for gene expression. Initial analyses focused on inverse correlations between methylation status (hypermethylated or hypomethylated) and gene expression.

Recent evidence supports the idea that morphologic and molecular signatures between HGSOC and normal fallopian tube are more similar than between HGSOC and normal ovarian epithelium^31–36^. Thus, we examined differential methylation patterns between normal fallopian tubes and primary HGSOC tumors. Patient characteristics and distribution of controls and samples for the study are provided in Table 1 and Fig. 1. Out of the ~850,000 methylation CpG probes in the Illumina Infinium MethylationEPIC Array, there are 66,069 probes that interrogate CpG sites of all known genes as categorized by Illumina. Of these 66,069 probes, 5,852 probes are significantly different between the fallopian tube controls and HGSOC. For this comparison, significance is defined by a p-value of 10^−7^ and >2 fold change difference (Fig. 2A). When the genes represented by the probes that are different between the controls and HGSOC are plugged in to the KEGG wiring diagram database where genes are integrated into a pathway map and analyzed, several pathways are noted to be enriched, such as the RAS, dopaminergic synapse, Adherens junction, and Hippo signaling pathways. Other interesting pathways involve dopaminergic synapse, axon guidance, and GABAergic synapse signaling (Fig. 2B).

We next looked at the correlation between methylation status and gene expression. The 5,852 differentially methylated probes comparing HGSOC and fallopian tubes in our UIHC cohort represent 4,593 genes of which 725 are hypomethylated and 3,868 are hypermethylated. If hypomethylation directs up-regulation of gene expression and hypermethylation directs down-regulation of gene expression then an inverse relationship between methylation and gene expression should be meaningful. In our data 175 hypomethylated genes displayed higher expression and 1,173 hypermethylated genes displayed lower expression (Fig. 2C). This represents 24% and 30% of genes respectively. Thus, only about a quarter of differentially methylated loci show differences in expression consistent with a cis-acting factor.

**Figure 3:**
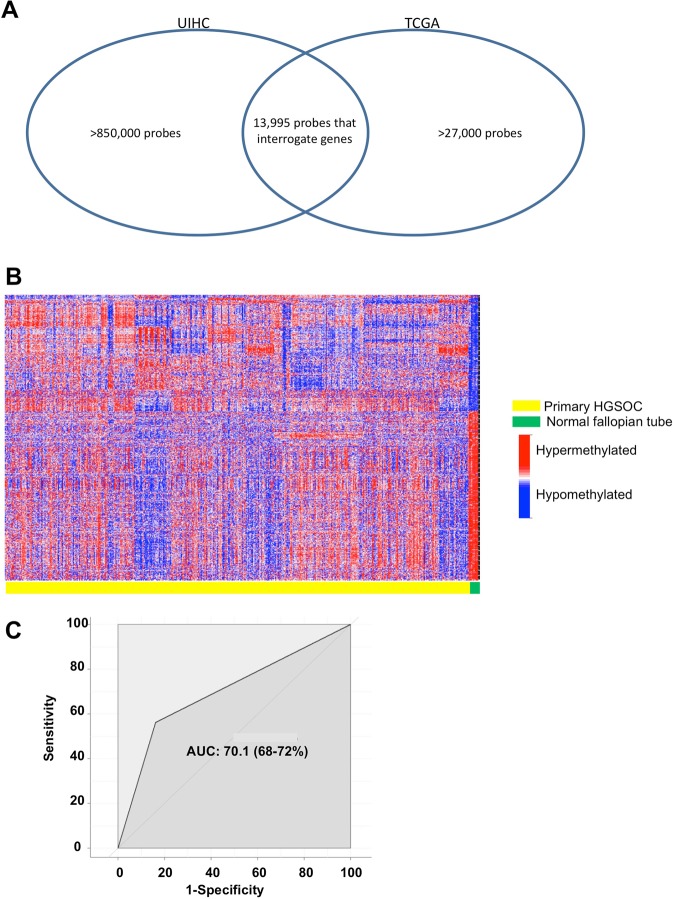
Validation of results using the Cancer Genome Atlas (TCGA) dataset. (**A)** Venn diagram showing the overlap in CpG methylation probes used in the UIHC and TCGA cohorts. (**B**) Heat map clustering of shared probes between UIHC and TCGA datasets. Each column represents a patient sample and each row represents a probe that interrogates CpG sites of known genes as categorized by Illumina. (**C**) Receiver operating characteristic curve showing the degree of agreement via AUC between UIHC and TCGA data when looking at significantly different methylation probes between normal and HGSOC. AUC, area under the curve.

To put the above results in context we imported HGSOC and normal fallopian tube methylation data from TCGA (Fig. 1C) and performed the same analysis. As noted above, TCGA used the Infinium HumanMethylation27 BeadChip assay, which is an earlier version of the array containing 27,578 probes compared with the >850,000 probes in the Infinium MethylationEPIC BeadChip used by us. We identified 13,995 probes interrogating genes sharing the same nomenclature in both versions of the platform (Fig. 3A). Out of 13,995 methylation CpG probes for known genes shared by both arrays, we found that 2,075 probes were significantly different between normal fallopian tubes and HGSOC in the TCGA data (Fig. 3B). When we compared TCGA results with our data, we see that there are 1,891 of 2,075 probes (91.1%) significant in both data sets. When we align our data against TCGA data, we see a substantial degree of agreement with an area under the curve (AUC) of 70.1% (Fig. 3C).

### Differential DNA methylation between primary and recurrent HGSOC

**Figure 4:**
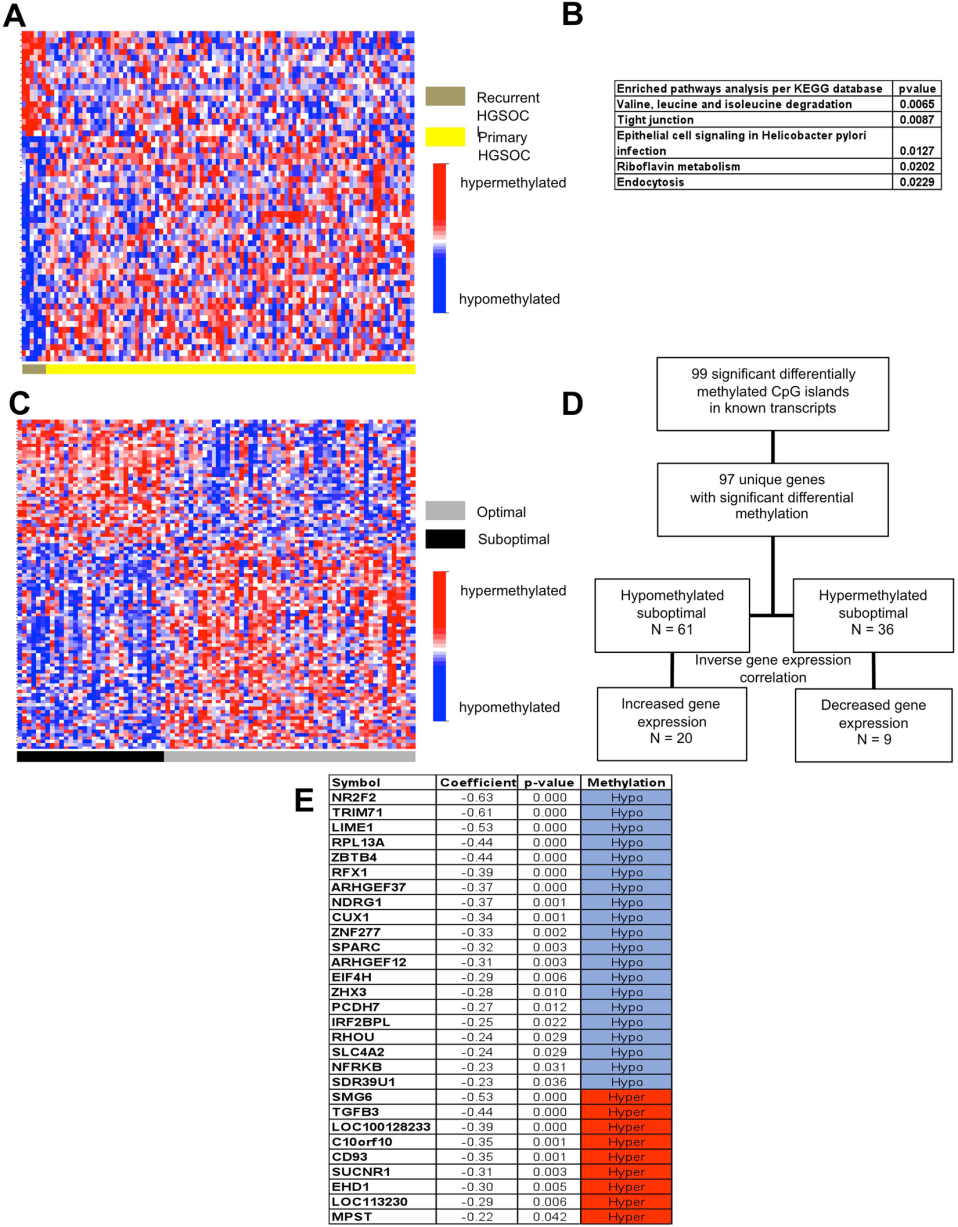
Differential methylation between primary versus recurrent high-grade serous ovarian cancer, and optimal versus suboptimal surgical outcomes. (**A**) Heat map of methylation probes that interrogating known genes in primary and recurrent high-grade serous ovarian cancer (HGSOC). (**B**) Pathways significantly enriched with genes that are differentially methylated between primary and recurrent HGSOC in the KEGG database. (**C**) Heat map of methylation probes that interrogating known genes for optimal versus suboptimal surgical outcomes in primary HGSOC. Optimal outcomes defines as residual disease of <1 cm, survival of >90 days after surgery, and chemotherapy administration within 8 weeks after surgery. (**D**) Experimental flow showing gene expression for probes representing specific loci differentially methylated between optimal and suboptimal surgical outcomes in primary HGSOC. Initial analysis focused on inverse correlations between methylation status (hypermethylated or hypomethylated) and degree of gene expression (increased or decreased). (**E**) List of genes presenting inverse correlations between methylation status and degree of gene expression.

Of the 66,069 probes interrogating CpG sites of known genes categorized by Illumina, 57 probes present significantly different methylation between primary and recurrent HGSOC (Fig. 4A). Genes represented by the differentially methylated probes enrich a number of pathways in the KEGG database (Fig. 4B). Among these are degradation of the amino acids valine, leucine and isoleucine, all aliphatic essential amino acids. Another enriched KEGG pathway is the riboflavin-vitamin B2 metabolism.

### Differential DNA methylation between optimal and suboptimal surgical outcomes

We also evaluated differential methylation patterns between patients judged to have had optimal versus suboptimal surgical outcomes. An optimal surgical outcome is agreed to have less than one centimeter to zero residual disease, survival of at least 90 days following surgery, and the ability tolerate chemotherapy within 8 weeks. Between our optimal and suboptimal surgical cases we observed 99 significant differential methylation events among 66,069 gene-associated probes (Fig. 4C). During pathway analysis, genes that are involved in cancer and in the interaction of infections such as tuberculosis and toxoplasmosis with the innate immune system such as transforming growth factor beta (TGFβ) and toll like receptor 4 (TLR4) were found to be differentially methylated (data not shown). Other enriched pathways include the apelin and hippo signaling pathways and metabolism of the sulphur-containing amino acids cysteine and methionine (data not shown).

We examined the correlation of methylation status and gene expression in these cases as well. The 99 probes interrogating genes that are significantly different between optimal and suboptimal cases identified 97 genes. Of these 97 genes, 61 are hypomethylated and 36 are hypermethylated in suboptimal cases. Inverse relationships between methylation status and gene expression were observed for 20 (33%) upregulated out of 61 hypomethylated genes and 9 (17%) downregulated out of 36 hypermethylated genes (Fig. 4D). A complete list of the 29 genes that showed the expected inverse correlation between methylation status and gene expression are shown in Fig. 4E.

### Overall methylation pattern of HGSOC

**Figure 5:**
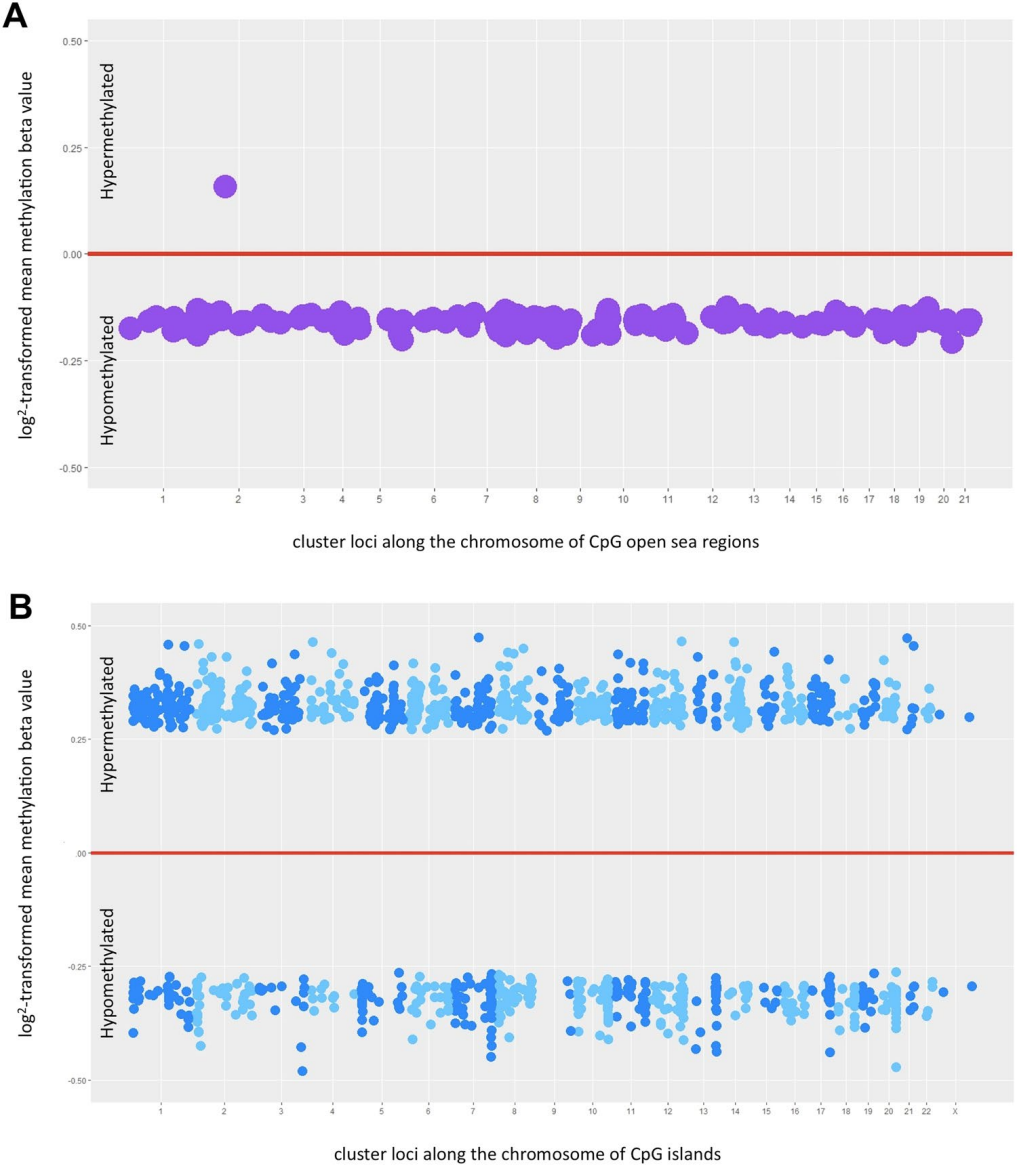
Global methylation status and promoter methylation status in high-grade serous ovarian cancer. (**A**) Global methylation status was evaluated using probes that identify isolated loci of methylated CpGs in the open sea regions of the genome defined as >4 Kb away from the nearest CpG island. Each dot represents a cluster of long methylated DNA segments (**B**). Promoter methylation status was evaluated using probes that identify shorter genomic regions of CpGs. Each dot represents a cluster of contiguous CpG regions knows as CpG islands and/or shorelines. The alternating shades of color are used to differentiate a cluster located in one chromosome from another in a different chromosome. In (**A**,**B**) the numbers on the X- axis represent chromosomes. The dots appearing above the red line represent hypermethylated regions, while those appearing below are hypomethylated.

Using probes that detect long-range clusters of CpGs, so-called “open seas,” global methylation status between normal fallopian tube and HGSOC in the UIHC cohort showed that 154 out of 1,058 long-range methylated regions are significantly different (p-value < 10^−3^), with an average size of 701 Kb. All but one of the probes detected hypomethylation in HGSOC compared with fallopian tubes. That one exception is a region of chromosome 2 that is hypermethylated (Fig. 5A and Supplementary Table 1).

When probes that detect clusters of contiguous CpGs were used, 1,408 out of 17,847 clusters of CpG regions were significantly different (accounting for multiple comparisons: p-value < 10^−4^), with each cluster having an average size of 0.21 Kb. These contiguous clusters, or “shorelines,” of CpGs are almost exclusively located in promoter regions of known genes and tend to be hypermethylated in HGSOC compared to normal fallopian tubes (Fig. 5B and Supplementary Table 2).

## Discussion

Despite improved understanding of the tumor microenvironment and genomic heterogeneity coupled with numerous clinical trials investigating novel treatment agents, the 5-year relative survival rate of around 30–40% for advanced ovarian cancer has barely improved over the past two decades^8^. Epigenetic regulation has recently emerged as an important additional mechanism affecting ovarian cancer and adding another dimension to our ability to sub-classify patients and customize their treatment^37^.

To our knowledge, our panel is the largest cohort of HGSOC samples examined to date using the most comprehensive collection of methylation probes, the MethylationEPIC array (850,000 probes), to perform an epigenome-wide comparative methylation association study. In our analysis of overall DNA methylation, we observed two different methylation patterns that depend upon the CpG sites being interrogated and the probes used. When considering global methylation status detected by long-range CpG probes that include CpG sites in both island and open sea regions^28^, there is nearly universal hypomethylation in HGSOC compared with fallopian tube controls (Fig. 5A). When considering probes interrogating CpG islands and shorelines located around gene promoter regions, there is a mixture of methylation patterns with a higher proportion of hypermethylated sites (Fig. 5B). This observation is consistent with previous studies suggesting overall DNA hypomethylation affecting oncogenes and a more specific hypermethylation of tumor suppressor gene promoters^23^. Bartlett *et al*. primarily looked at epigenetic differences between proximal and distal fimbrial segments of normal fallopian tubes in patients with BRCA mutations and controls. Although their study is different from ours, they found that the significant differential methylation levels are threefold higher in BRCA mutation carriers than in controls with the fimbriated segments showing 81% hypomethylation and 19% hypermethylation on CpGs with a 40.03 difference in median beta value compared to proximal segments. Pisanic *et al*. also found that in a cohort that included 11 normal fallopian tubes, 23 HGSOC, and 9 serous tubal intraepithelial carcinoma (STIC) lesions, the location where the significant majority of the discrepancy in methylation levels between cancerous versus healthy tissues are not in the CpG islands but rather are in the CpG shores, shelves and open sea regions consistent with what we found^38,39^.

Focusing on the differential methylation profile between normal fallopian tube and HGSOC, we identified 5,852 probes interrogating known genes (Fig. 2A). This has potential implications for screening those at risk of developing HGSOC. DNA methylation changes are thought to occur early in tumors, contributing to carcinogenesis, and thus have potential use as screening biomarker panels. Pisanic *et al*. previously identified a panel of three genes (c17orf64, IRX2, and TUBB6) with methylation patterns that discriminate among normal fallopian tubes, STIC, and HGSOC^38^. Other investigators have reported on methylation biomarkers that identify HGSOC. Several studies identifying genes exhibiting promoter hypermethylation (E-cadherin or CDH1, H-cadherin, BRCA1, RASSF1A, APC, p14 ARF, p16INK4A, DAPK, RUNX3, TFPI2, SFRP5, and OPCM) have better sensitivity and specificity than CA-125, with some even detectable in serum or plasma^23,40^. We did not specifically evaluate all of these genes but did investigate some of them and found similar promoter hypermethylation in CDH1, BRCA1, and RUNX3 while APC was hypomethylated in our HGSOC cohort compared to controls.

We also examined differential methylation patterns between primary and recurrent HGSOC tissues, as well as among patients who had optimal vs suboptimal surgical outcomes as defined above. We believe these two comparison groups can serve as surrogates for biologic differences making tumors behave more aggressively and therefore more likely to recur or less likely to be optimally/completely cytoreduced during surgery. Clearly, more aggressive tumors decrease the likelihood of complete cytoreduction and increase the likelihood of subsequent recurrence of disease. Promoter hypermethylation has previously been correlated with early recurrence^41^. A study using 40 ovarian cancer patients divided into groups of short vs long progression free survival (PFS) identified prognostic DNA methylation profiles with 112 methylated loci prognostic for PFS in advanced ovarian cancer patients^42^. Hypermethylation of specific loci such as HOXA9 and BRCA is more common in serous compared to other histologic subtypes, and has been associated with decreased expression and advanced stage ovarian cancers compared to benign or stage I cases^23^. While certain drivers of tumorigenesis such as LINE-1 can be hypomethylated and portends poor prognosis^43^, some immune response genes can undergo hypomethylation leading to improved tumor infiltration of CD8 T-cells, and longer time to recurrence^44^. Our data revealed 57 differentially methylated probes between primary vs recurrent disease (Fig. 4A) that can potentially be used as biomarkers to identify those patients who may benefit from a novel maintenance treatment regimen. However, we must caution that, though this result is statistically significant, our recurrence group does consist of just six cases (Fig. 1B).

Our study is the first to examine methylation status in relation to surgical outcomes. Here we had 54 patients (62.8%) whose surgeries were considered to be optimal and 32 patients (37.2%) whose surgeries were considered to be sub-optimal. We identified a total of 97 loci with significant differential methylation between our optimal and suboptimal cases. These loci, in combination with other clinical variables, can be used to predict who will benefit from neoadjuvant chemotherapy or novel targeted treatment regimens informed by their surgical outcome.

Epigenetic signatures identify specific loci and these loci can be grouped into relevant ontologic pathways that can, in turn, be useful in identifying drivers influencing the behavior of HGSOC. Using loci identified in our differential methylation study, pathway analyses indicate that RAS signaling is the most enriched pathway in HGSOC tumor tissues compared with benign fallopian tube tissues (Fig. 2B). Methylation of genes involved in the RAS signaling pathway, such as the RASSF1A gene, have previously been reported in ovarian cancer^45^. Our result is consistent with the previously reported finding that RAS is associated with epigenetic regulation in ovarian cancer via DNMT enzyme activity^46^. Other enriched pathways identified include dopaminergic synapse, axon guidance, and GABAergic synapse among others. Interestingly, recent interest in autonomic innervation as an important component of the tumor microenvironment has been suggested to contribute to cancer initiation and metastasis^47–49^. Meanwhile, for patients with recurrent disease compared with primary disease, the most enriched pathway is degradation of the aliphatic essential amino acids valine, leucine, and isoleucine (Fig. 4B). This pathway is intimately involved in mitochondrial degradation via autophagy (mitophagy) and has previously been linked to invasion and metastasis in epithelial ovarian cancers^50^. In addition, the tight junction pathway and, in particular, the Claudin gene family has been implicated in HGSOC recurrence^51^.

With regard to our examination of optimal and suboptimal surgical outcomes, the two genes that are prominently identified are the immune response drivers TGFβ and TLR4. Both of these genes are also well known genes involved in cancer^52^. TLR4 encodes a transmembrane protein whose activation leads to the intracellular signaling pathway of nuclear factor-kappa B (NFκB) and to immune-related cytokine production responsible for activating the innate immune system. There are reports that TLR4 can regulate response to paclitaxel in ovarian cancer^53^ while other studies showed no association with survival in HGSOC^54^. Secreted protein acidic and rich in cysteine (SPARC) is another interesting gene that showed hypomethylation in suboptimal vs optimal surgical outcomes sub-group with concomitant upregulation of gene expression (Fig. 4D,E). SPARC is a membrane-associated glycoprotein that binds calcium whose overexpression has been previously associated with a more invasive, higher stage, and poorer prognosis in a cohort of 140 ovarian tissue samples which include 80 tissues from invasive epithelial ovarian cancer patients consistent with our findings^55^. In contrast other studies have shown promoter hypermethylation and lower gene expression of SPARC being associated with HGSOC compared to controls^56^. This discrepancy may be related to several factors such as the ratio of stromal to epithelial components of the samples, or the effect of other closely related proteins within the SPARC family. Case in point, when we looked at SPARCL1 a member of the SPARC subfamily we noted hypermethylation and a 7-fold lower gene expression in primary HGSOC compared to controls (P < 0.01) consistent with the TCGA findings^15^.

Assuming that hypermethylation will lead to reduced gene expression and hypomethylation will lead to increased gene expression we looked at the correlation of methylation status and gene expression and focused on those with inverse relationships. Surprisingly, we found that only 17–33% of the genes met expected gene expression results based on their methylation status (Figs. 2C and 4D,E). Of note, even in TCGA HGSOC only 168 out of 14,475 probed genes were downregulated in conjunction with DNA hypermethylation^15^. Given these findings, we must conclude that methylation status does not necessarily lead to expected gene expression changes. Obviously, this may be due to other variables affecting gene expression. A gene can have multiple promoters, and if one promoter is methylated but a gene is dependent on a different promoter to drive it in a specific tissue or circumstance, then that gene will remain unaffected and still be transcribed. Other factors such as long non-coding RNAs, micro-RNAs, and structural changes in the DNA can also affect gene regulation. The incongruent relationship between methylation and expression can also be due to other less common modifications of histones like the monoubiquitination of histone H2B (H2Bub1) that regulates gene expression whose loss leads to an open chromatin state and upregulation of immune modulators. An assay for transposase-accessible chromatin using sequencing (ATAC-seq) could be employed on further studies to validate such mechanisms as done by Hooda *et al*.^57^.

In the future, a prediction model combining epigenetic signatures with other clinical and biologic variables may be able to better characterize patients into treatment groups. TCGA analyses designated four subtypes based on variable DNA methylation clusters but did not sort patients into who is more likely to recur or respond to a particular treatment regimen^15^. Epigenetic regulators may be used as targeted treatment for cases where DNA methylation is a primary driver of disease. Ushijima *et al*. noted that while genetic mutations are constant and set once they occur, epigenetic mechanisms such as DNA methylation remain dynamic and reversible^58^. The ability to affect the epigenetic silencing machinery makes it an ideal pharmacologic target with the potential to prevent and treat cancers^59^. Hypermethylation of loci SFRP5, MLH1, ASS1, MCJ, and ESR2 correlate with platinum resistance^60–64^ and are potentially reversible using epigenetic therapies that include DNMT inhibitors^23,65^. DNMT inhibitors like decitabine and azacytidine are nucleoside analogues that trap DNMTs and reverse hypermethylation of tumor suppressors, promoting their subsequent re-expression with continuous drug administration^21^. They have been used in ovarian cancer clinical trials and have shown response in previously platinum-resistant patients^66,67^, though some showed less promising results in the platinum-sensitive setting in combination with chemotherapy^68^.

A better understanding of how methylation of specific CpG promoter sites affects variation in gene expression and tumor behavior can be gained through cell culture experiments and mouse patient-derived xenograft (PDX) models. Promoter hypermethylation of BRCA1 has been observed in up to 20% of HGSOCs, though reports on its impact on overall survival have been mixed^15,69^. Questions relative to the impact of poly ADP-ribose polymerase (PARP) inhibitors on hypermethylated BRCA1 genes and other homologous recombination repair genes that are hypermethylated in HGSOC, such as RAD51C^15^, as well as the effect of immune checkpoint inhibitors on hypermethylated MLH1^70^, can be specifically assessed in the future.

Finally, in this study the stromal and epithelial composition of both the samples and controls were not quantified which may impact the results. In the future, an immunohistochemistry-based scoring system that addresses this limitation may be employed to reduce the impact of cellular distribution as a confounding variable. We used sections of fallopian tubes that included stromal components and not just the epithelial layer as controls. The TCGA used controls similar to ours, but we believe that a laser-dissected specimen that isolates the epithelium might have less potential biases^36^. The logistics of obtaining enough paired RNA and gDNA from isolated epithelium from fimbriated ends of the fallopian tube will be extremely challenging, however. We started with 20 samples and ended up with 12 controls that had enough high-quality RNA and gDNA to conduct all of the planned experiments. We also had enough HGSOC samples producing gDNA and RNA of sufficient quality to allow us to address not just disease versus controls but also the issues of recurrence and of surgical outcomes. Another limitation of the study is the absence of paired samples. Pisanic *et al*. found hypermethylated loci in STIC lesions that are absent in normal fallopian tubes. We believe that the addition of paired fallopian tube and tumor samples from patients could provide better insight in the progression of normal fallopian tube to STIC and HGSOC and how it differs to an unpaired normal fallopian tube. Matched samples are unavailable for the projects conducted by our and Pisanic’s group could be explored in the future^38^.

In conclusion, we found significant differences in methylation patterns between HGSOC and fallopian tube controls, primary vs recurrent tissues, and optimal vs suboptimal surgical outcomes. Some of these differences may be utilized to help identify candidate loci through further analyses of specific methylation/gene expression discordance. However, the low rate of concordance between methylation and gene expression in our data suggests that there is more complexity to be discovered. Cataloging these phenomena in a well-characterized clinical population will aid in determining appropriate groupings for screening and for precision medicine treatment cohorts in the future^18^.

## Supplementary information

Dataset 1

## Data Availability

We have submitted our DNA methylation data to the NCBI Gene Expression Omnibus and it is available under Accession Number GSE133556.
