# Supplementary material for: Differential DNA methylation in high-grade serous ovarian cancer (HGSOC) is associated with tumor behavior
Source: Sci Rep. 2019 Nov 29;9:17996. doi: 10.1038/s41598-019-54401-w (PMC6884482; doi:10.1038/s41598-019-54401-w)
Supplement: Supplementary file 1 — Dataset 1 [file 41598_2019_54401_MOESM1_ESM.pdf]

Differential DNA methylation in high-grade serous ovarian cancer (HGSOC) is associated with tumor behavior

Henry D. Reyes, Eric J. Devor, Akshaya Warrier, Andreea M. Newtson, Jordan Mattson, Vincent Wagner, Gabrielle N. Duncan, Kimberly K. Leslie, Jesus Gonzalez-Bosquet

Supplemental Table 1: Long-range "open seas" with significant differential methylation signatures. Negative methylation values represent hypo-methylation in HGSOC compared with fallopian tube controls. Chromosome coordinates are GRCh38 numbers.

Chromos	Start	End	Length (kb)	value	p-value
1	4886129	5295746	409.6	-0.17369	2.73E-05
1	71809745	72190606	380.9	-0.15652	3.00E-04
1	96144934	96629912	485.0	-0.14793	7.64E-04
1	158217942	158944784	726.8	-0.18	1.00E-08
1	159165562	159697240	531.7	-0.14994	2.73E-04
1	187343088	187840215	497.1	-0.17138	2.73E-05
1	240874827	241639410	764.6	-0.14343	4.09E-04
1	242187498	243100073	912.6	-0.18989	1.00E-08
1	243706206	244192443	486.2	-0.13064	9.83E-04
1	248159309	248907659	748.4	-0.14991	2.73E-05
2	1878311	2769039	890.7	-0.14173	8.19E-05
2	3786250	4306408	520.2	-0.14081	9.55E-04
2	13623703	15012948.5	1389.2	-0.16228	1.09E-04
2	21442643	22147895	705.3	-0.14921	3.00E-04
2	49980786	51013681	1032.9	-0.1362	7.91E-04
2	56968218	57771498	803.3	-0.15267	5.19E-04
2	66666971	67041254	374.3	0.15796	1.36E-04
2	116098870	116852238	753.4	-0.16414	1.36E-04
2	125195905	127315703	2119.8	-0.15826	5.46E-05
2	137516183	138045743	529.6	-0.15797	2.18E-04
2	199431605	200245170	813.6	-0.14346	7.10E-04
2	228855878	230179047	1323.2	-0.15675	5.46E-05
3	5796595	6832844	1036.2	-0.15979	1.64E-04
3	76292114	76645048	352.9	-0.15053	6.00E-04
3	84158113	85410842	1252.7	-0.14508	5.46E-04
3	115919469	116535204	615.7	-0.14228	7.91E-04
3	161884911	162135697	250.8	-0.15607	5.46E-04
3	179672470	180297596	625.1	-0.15416	1.91E-04
4	9336287.5	9371217.5	34.9	-0.16897	2.73E-04
4	11967137	12314075	346.9	-0.15494	3.00E-04
4	13767630	14390632	623.0	-0.13634	6.28E-04
4	29710263	29751733	41.5	-0.18738	5.46E-05
4	36641040	37020018	379.0	-0.15596	3.28E-04

4	45946199	46650206	704.0	-0.1708	5.46E-05
5	1892110	5166199.5	3274.1	-0.16634	1.00E-08
5	5535886	6544833	1008.9	-0.15193	2.73E-05
5	7364007	7794275	430.3	-0.15961	1.64E-04
5	11774991	11931819	156.8	-0.17314	8.19E-05
5	113588550	114026851	438.3	-0.15341	2.73E-04
5	152234077	152591026	356.9	-0.18674	1.00E-08
5	152805675	153285036	479.4	-0.1667	2.73E-05
5	161855622	162051083	195.5	-0.20147	1.00E-08
5	163060530	167396870	4336.3	-0.15634	1.00E-08
6	47778694	48971322	1192.6	-0.15637	1.64E-04
6	76782041	77325193	543.2	-0.15087	4.91E-04
6	123343266	123597874	254.6	-0.15867	3.82E-04
6	165821961	166417490	595.5	-0.14501	1.09E-04
7	14165745	14353119.5	187.4	-0.16919	1.64E-04
7	31827390	32315577	488.2	-0.14045	6.00E-04
7	108486939	109096260	609.3	-0.15582	4.91E-04
7	113517552	113952882	435.3	-0.1694	5.46E-05
7	118372311	118498886	126.6	-0.1577	8.46E-04
7	119112663	119260946	148.3	-0.17894	1.00E-08
7	125942245	126496590	554.3	-0.14461	9.01E-04
7	126675358	126884639	209.3	-0.15338	4.09E-04
7	140739534	141190187	450.7	-0.13886	7.64E-04
7	141564636	141999418	434.8	-0.13193	9.28E-04
7	144604903	145450472	845.6	-0.19147	1.00E-08
7	145945578	146130429	184.9	-0.17066	3.00E-04
7	146720272	147849501	1129.2	-0.14559	4.37E-04
7	153754822	154651619	896.8	-0.13565	4.09E-04
7	157255232	158336690	1081.5	-0.17184	1.00E-08
8	798432	1645045	846.6	-0.14425	1.00E-08
8	2009485	3102057.5	1092.6	-0.14679	2.73E-05
8	3233045	4329150	1096.1	-0.14812	3.00E-04
8	4504923.5	5028854	523.9	-0.1686	2.73E-05
8	5367248	6038136	670.9	-0.14892	4.91E-04
8	13707000.5	14018761	311.8	-0.17637	2.73E-05
8	34280422	36235724	1955.3	-0.18573	1.00E-08
8	49846578	51364244	1517.7	-0.15493	2.73E-05
8	51926793	52365888	439.1	-0.16899	5.46E-05
8	62788052	63147364	359.3	-0.14736	7.10E-04
8	65462682	66131741	669.1	-0.16769	2.73E-05
8	76227159	76560371	333.2	-0.14892	8.46E-04
8	85560350	85832904	272.6	-0.17024	5.46E-05
8	109009486	109145172	135.7	-0.15895	3.00E-04
8	111620462	111719992	99.5	-0.1793	1.09E-04
8	113475312	114115265	640.0	-0.15118	4.64E-04
8	131761856	132594841	833.0	-0.14695	4.64E-04
8	135935344	136409523	474.2	-0.16822	5.46E-05

8	137619505	138231660	612.2	-0.17272	1.00E-08
8	138738134	139165566	427.4	-0.19621	1.00E-08
8	140035732	140659783	624.1	-0.15259	2.18E-04
9	10366759	10633641	266.9	-0.18758	1.00E-08
9	23672559.5	24920862	1248.3	-0.16181	1.36E-04
9	25261450	26502756	1241.3	-0.15482	2.73E-04
9	104473642	104757854	284.2	-0.18912	1.00E-08
9	119691550	120210352	518.8	-0.18058	1.00E-08
9	121181018	121505108	324.1	-0.17697	2.73E-05
10	1547721	3015699.5	1468.0	-0.16063	1.00E-08
10	7049378	7477590	428.2	-0.14354	2.73E-04
10	9999498	11272899	1273.4	-0.13016	1.64E-04
10	16032954	16441897	408.9	-0.18741	1.00E-08
10	18124504	18385632	261.1	-0.15176	4.37E-04
10	106232717	107597533	1364.8	-0.15466	1.36E-04
10	107935977	108342212	406.2	-0.14773	6.28E-04
10	108855141	110214215	1359.1	-0.14949	3.00E-04
10	110303937	110701934	398.0	-0.16375	1.36E-04
10	130040865	130659198	618.3	-0.14728	1.36E-04
10	132177258	133648934	1471.7	-0.14374	1.00E-08
11	4713544	5552785	839.2	-0.1668	1.00E-08
11	24436937	24849257	412.3	-0.15912	3.00E-04
11	42862161	43168034	305.9	-0.17017	5.46E-05
11	55338135	56587403.5	1249.3	-0.17735	1.00E-08
11	57881602	58192062	310.5	-0.1473	5.46E-04
11	59659419	60296549	637.1	-0.16174	1.00E-08
11	83153008	83436492.5	283.5	-0.14166	7.37E-04
11	87224821	87434057	209.2	-0.15126	9.55E-04
11	131533252	132891713	1358.5	-0.18474	1.00E-08
12	61899255	62512367	613.1	-0.14714	7.10E-04
12	103437918	103787156	349.2	-0.1613	1.91E-04
12	115468491	116291234	822.7	-0.12532	7.64E-04
12	118955657	119347187	391.5	-0.15951	1.64E-04
12	126520740	127483006	962.3	-0.13385	4.37E-04
12	129415102	130446362	1031.3	-0.15264	2.73E-05
13	26051676	26564488	512.8	-0.14441	3.82E-04
13	56511952	57133493	621.5	-0.1643	1.36E-04
13	61455634	61660030	204.4	-0.16523	2.46E-04
13	107910972	108657826	746.9	-0.1695	1.00E-08
13	109071839	109387041	315.2	-0.15279	4.09E-04
13	112082093	113088886	1006.8	-0.16896	1.00E-08
14	27545522	27836139	290.6	-0.16088	8.19E-04
14	87716101	87897658	181.6	-0.17004	5.46E-05
15	26447291	27035142.5	587.9	-0.15985	1.64E-04
15	37697375	37974776	277.4	-0.16233	7.64E-04
15	53252822	53485623	232.8	-0.1638	2.18E-04
16	5373567	6661697	1288.1	-0.15149	5.46E-05

16	17054840	17685871	631.0	-0.135	1.36E-04
16	17834936	18246884	411.9	-0.15105	2.46E-04
16	25586856	25851007	264.2	-0.15396	4.09E-04
16	48865450	49204333	338.9	-0.14865	4.91E-04
16	60448068	61084547	636.5	-0.1503	9.83E-04
16	76296523	77044480	748.0	-0.15017	2.46E-04
16	82314773	83803650	1488.9	-0.16801	1.00E-08
17	49513053.5	51063065	1550.0	-0.15369	1.00E-08
18	4106506	4599319	492.8	-0.18323	1.00E-08
18	4756135	5232798	476.7	-0.16391	1.36E-04
18	5393019	5747675.5	354.7	-0.15721	2.18E-04
18	66504307.5	67432783	928.5	-0.15701	5.46E-05
18	68664091	69065767	401.7	-0.14733	9.55E-04
18	70339257	71563310	1224.1	-0.14599	1.64E-04
18	75010803	76760253	1749.5	-0.18836	1.00E-08
19	30596691.5	31155284	558.6	-0.14118	5.19E-04
19	31939554	32403368	463.8	-0.15777	3.00E-04
19	54989027	55283829	294.8	-0.12741	9.01E-04
19	56288909.5	56549544	260.6	-0.14977	5.73E-04
20	6246047	7373216	1127.2	-0.15755	2.73E-05
20	40490942	41319967	829.0	-0.15524	1.64E-04
20	41574350	41952073	377.7	-0.16917	5.46E-05
20	58755997	60489355	1733.4	-0.2065	1.00E-08
21	22696706	23493155	796.4	-0.15524	2.18E-04
21	31579225	32334697	755.5	-0.16687	1.00E-08
21	41706246	42259157	552.9	-0.15551	1.64E-04
X	86919857	86919857	0.0	1.01752	6.28E-04

Supplemental Table 2: Short-range "shores" with significant differential methylation signatures. Positive methylation values indicate hyper-methylation in HGSOC compared with fallopian tube controls and negative methylation values indicate hypomethylation in HGSOC compared with fallopian tube controls.
Chromosome coordinates are GRCh38 numbers.

Chromos	start	end	Length (kb)	value	p-value
chr1	1297567	1297576	0.01	0.32022	0
chr1	1566351	1566699	0.35	0.29097	0
chr1	2006032	2006139	0.11	0.31908	0
chr1	2205770	2205902	0.13	0.34635	0
chr1	2425860	2425952	0.09	-0.30717	0
chr1	2918724	2918752	0.03	-0.32131	0
chr1	3147622	3147966	0.34	-0.39671	0
chr1	3809431	3809532	0.1	0.32626	0
chr1	4221401	4221485	0.08	-0.29494	0.00064
chr1	4663943	4664431	0.49	-0.28549	0.00042

chr1	5062141	5062216	0.08	-0.34356	0
chr1	6336519	6336743	0.22	0.31057	0
chr1	7913067	7913579	0.51	0.36023	0
chr1	8271918	8272081	0.16	-0.30581	0
chr1	8651018	8651231	0.21	0.29325	0.00085
chr1	10292174	10292195	0.02	0.3386	0
chr1	12164457	12164477	0.02	0.32587	0
chr1	12493605	12493840	0.24	0.30816	0
chr1	16847157	16847214	0.06	0.32108	0
chr1	18686618	18687127	0.51	-0.29188	0
chr1	22416016	22416488	0.47	0.30685	0
chr1	24152579	24152774	0.2	-0.32512	0
chr1	24648203	24648328	0.13	-0.32297	0
chr1	25254129	25254149	0.02	0.29857	0.00021
chr1	25893405	25893744	0.34	0.29929	0
chr1	27189985	27190330	0.35	-0.30116	0
chr1	28843701	28843736	0.04	-0.31924	0
chr1	33231178	33231190	0.01	0.30303	0.00021
chr1	33803805	33804213	0.41	0.32852	0
chr1	34175120	34175317	0.2	-0.2825	0.00021
chr1	38481765	38482118	0.35	0.34725	0
chr1	39724134	39724195	0.06	0.30285	0.00021
chr1	41454155	41454277	0.12	0.307	0
chr1	43609431	43609461	0.03	0.33949	0
chr1	47694919	47695138	0.22	0.33601	0
chr1	47698493	47698976	0.48	0.29322	0
chr1	55013735	55013956	0.22	0.31	0
chr1	55794598	55794878	0.28	0.32516	0
chr1	56975583	56975689	0.11	0.3221	0
chr1	59484600	59484803	0.2	0.28444	0.00042
chr1	61586157	61586195	0.04	0.32404	0
chr1	62438955	62439021	0.07	0.34074	0
chr1	62660435	62660624	0.19	0.35497	0
chr1	62660850	62660861	0.01	0.32059	0
chr1	72382645	72382835	0.19	-0.312	0
chr1	84629943	84630050	0.11	0.29557	0
chr1	89357945	89357988	0.04	-0.31288	0
chr1	91185233	91185422	0.19	0.29777	0.00021
chr1	91185749	91185922	0.17	0.31264	0
chr1	91188999	91189167	0.17	0.29898	0.00021
chr1	91300288	91300390	0.1	0.27801	0.00042
chr1	94079684	94079866	0.18	0.33061	0
chr1	95123877	95124061	0.18	0.3613	0
chr1	95507935	95508282	0.35	0.30632	0
chr1	109506658	109506696	0.04	-0.30326	0.00021
chr1	110254828	110254835	0.01	0.34857	0
chr1	111970925	111971020	0.1	0.28661	0.00021

chr1	114489550	114489995	0.45	0.34261	0
chr1	114842021	114842468	0.45	0.31408	0
chr1	116925194	116925753	0.56	0.34336	0
chr1	118389869	118390138	0.27	0.39743	0
chr1	119526060	119526255	0.2	0.39131	0
chr1	147781212	147781600	0.39	0.33669	0
chr1	147781827	147782558	0.73	0.33182	0
chr1	147790458	147790806	0.35	0.32381	0
chr1	150448887	150448943	0.06	0.3846	0
chr1	150779369	150780019	0.65	0.34382	0
chr1	150780684	150781434	0.75	0.35407	0
chr1	152197889	152198107	0.22	-0.3022	0.00021
chr1	153363020	153363264	0.24	-0.29353	0.00064
chr1	153724779	153725318	0.54	0.37302	0
chr1	154407010	154407027	0.02	0.30199	0.00021
chr1	154407366	154407478	0.11	0.45912	0
chr1	154929455	154929657	0.2	0.31216	0
chr1	154942566	154942910	0.34	0.32446	0
chr1	155188904	155188982	0.08	0.30195	0.00021
chr1	158549277	158549410	0.13	-0.33434	0
chr1	158564533	158564883	0.35	-0.32509	0
chr1	158604822	158605757	0.94	-0.31217	0
chr1	158656484	158656718	0.23	-0.31761	0
chr1	158670617	158670761	0.14	-0.3046	0.00021
chr1	159283536	159284298	0.76	-0.27318	0.00021
chr1	160992119	160992358	0.24	-0.27239	0.00085
chr1	162518082	162518158	0.08	0.3234	0
chr1	162601805	162601807	0	0.32481	0
chr1	164544496	164544748	0.25	0.30837	0
chr1	164545553	164545783	0.23	0.3183	0
chr1	164595186	164595269	0.08	0.27618	0.00042
chr1	164681588	164681652	0.06	0.29038	0
chr1	164744047	164744436	0.39	0.31138	0
chr1	165631596	165631946	0.35	0.30701	0
chr1	166459325	166459524	0.2	-0.31631	0
chr1	167397535	167397908	0.37	0.32524	0
chr1	170638807	170639289	0.48	0.34447	0
chr1	171226993	171227124	0.13	0.28012	0.00042
chr1	172113506	172114857	1.35	0.32762	0
chr1	174968015	174968144	0.13	0.32611	0
chr1	181767736	181767774	0.04	-0.31465	0
chr1	183107083	183107206	0.12	0.30893	0
chr1	187610300	187610488	0.19	-0.29049	0
chr1	201465777	201465789	0.01	0.35318	0
chr1	201982886	201982957	0.07	-0.32529	0
chr1	202379967	202379988	0.02	0.36693	0
chr1	208012106	208012118	0.01	0.29498	0.00064

chr1	210468441	210468650	0.21	0.3201	0
chr1	210501621	210501634	0.01	0.29686	0.00042
chr1	212457424	212457538	0.11	-0.3545	0
chr1	215178601	215178658	0.06	0.33375	0
chr1	221054365	221054430	0.07	0.27638	0.00042
chr1	221055097	221055665	0.57	0.32873	0
chr1	221068423	221069136	0.71	0.33552	0
chr1	225767995	225768233	0.24	0.332	0
chr1	225768784	225769034	0.25	0.45597	0
chr1	226099128	226099147	0.02	0.31575	0
chr1	226862907	226862915	0.01	0.30087	0.00021
chr1	227962723	227963171	0.45	0.3069	0
chr1	234667230	234667363	0.13	0.3372	0
chr1	234908226	234908381	0.16	-0.30491	0.00021
chr1	242351909	242352195	0.29	-0.36608	0
chr1	242767727	242767942	0.22	-0.38332	0
chr1	245499492	245499541	0.05	-0.35732	0
chr1	246743272	246743403	0.13	0.35456	0
chr1	247511364	247511469	0.11	0.31622	0
chr1	248902767	248903100	0.33	-0.32718	0
chr2	468413	468989	0.58	0.30625	0
chr2	875509	875990	0.48	-0.28485	0.00042
chr2	1926535	1926724	0.19	-0.35228	0
chr2	2030359	2030712	0.35	-0.3011	0.00021
chr2	2193511	2193537	0.03	-0.35773	0
chr2	2321772	2321788	0.02	-0.3553	0
chr2	2334766	2334985	0.22	-0.29272	0.00085
chr2	2402353	2402636	0.28	-0.2948	0.00064
chr2	2692854	2692889	0.04	-0.37251	0
chr2	3633407	3633519	0.11	0.37131	0
chr2	3646077	3646196	0.12	-0.31377	0
chr2	3826621	3826631	0.01	-0.31272	0
chr2	4050695	4050845	0.15	-0.36021	0
chr2	8978201	8978225	0.02	-0.39509	0
chr2	9526703	9527051	0.35	-0.39274	0
chr2	10266949	10266986	0.04	0.46046	0
chr2	11606881	11607106	0.23	-0.33448	0
chr2	12443300	12443780	0.48	-0.31545	0
chr2	16804409	16804452	0.04	-0.42433	0
chr2	20650578	20650792	0.21	0.28655	0.00021
chr2	21618087	21618766	0.68	-0.27298	0.00085
chr2	23840537	23840739	0.2	0.2804	0.00042
chr2	27301195	27301369	0.17	0.27867	0.00042
chr2	27301490	27301651	0.16	0.3036	0
chr2	28182529	28182570	0.04	0.40105	0
chr2	28491946	28492416	0.47	0.32055	0
chr2	28857916	28858407	0.49	0.41874	0

chr2	38300537	38300707	0.17	0.28747	0.00021
chr2	38334178	38334245	0.07	0.29354	0.00064
chr2	38947733	38947762	0.03	0.32598	0
chr2	39621470	39621862	0.39	0.3056	0
chr2	40447561	40447599	0.04	0.29537	0.00064
chr2	40739515	40739771	0.26	0.30785	0
chr2	42175341	42175351	0.01	0.33511	0
chr2	45028225	45028269	0.04	0.29144	0
chr2	45159434	45159504	0.07	0.29735	0
chr2	45159663	45160490	0.83	0.29869	0
chr2	45395194	45395252	0.06	0.37989	0
chr2	45998165	45998548	0.38	0.29172	0.00085
chr2	46747628	46747951	0.32	0.30255	0.00021
chr2	46997959	46998070	0.11	0.34226	0
chr2	48737157	48737604	0.45	0.33037	0
chr2	55203963	55204245	0.28	0.34568	0
chr2	55339820	55339939	0.12	0.33698	0
chr2	62889846	62889918	0.07	0.50123	0
chr2	66466834	66466894	0.06	0.30888	0
chr2	66687552	66687704	0.15	0.29215	0.00085
chr2	66809959	66809985	0.03	0.29607	0.00064
chr2	66916716	66916737	0.02	0.38783	0
chr2	66918213	66918305	0.09	0.4318	0
chr2	67550622	67551130	0.51	0.37212	0
chr2	67939740	67939758	0.02	0.30237	0.00021
chr2	70322323	70322610	0.29	0.33906	0
chr2	71126275	71126505	0.23	0.32013	0
chr2	71133502	71133854	0.35	0.35015	0
chr2	74663416	74663482	0.07	0.29718	0.00042
chr2	74693332	74693344	0.01	-0.30264	0.00021
chr2	75767474	75767731	0.26	0.35584	0
chr2	85751446	85751463	0.02	0.32473	0
chr2	86263224	86263270	0.05	0.29685	0.00042
chr2	88367137	88367362	0.23	0.2733	0.00064
chr2	100722680	100723110	0.43	0.33523	0
chr2	101434082	101434121	0.04	-0.30948	0
chr2	101541372	101541592	0.22	0.36568	0
chr2	102445604	102445628	0.02	0.3767	0
chr2	103131227	103131398	0.17	-0.30248	0.00021
chr2	105216040	105216370	0.33	-0.30147	0.00021
chr2	105735617	105735756	0.14	0.30691	0
chr2	106013169	106013556	0.39	-0.30864	0
chr2	106755481	106755487	0.01	0.34754	0
chr2	113404661	113405125	0.46	-0.29809	0
chr2	127955156	127955187	0.03	-0.33573	0
chr2	130515726	130515731	0.01	0.30042	0.00021
chr2	131046480	131046551	0.07	-0.31586	0

chr2	131094827	131094852	0.03	0.43168	0
chr2	137747619	137747719	0.1	-0.29687	0.00042
chr2	138721433	138721454	0.02	0.30526	0.00021
chr2	144692441	144692640	0.2	0.30353	0.00021
chr2	162282922	162283189	0.27	0.29779	0.00021
chr2	169653213	169653384	0.17	0.3022	0
chr2	171784674	171784945	0.27	-0.35593	0
chr2	172972840	172973241	0.4	0.31215	0
chr2	172974138	172974217	0.08	0.30033	0.00021
chr2	173792831	173792840	0.01	0.34096	0
chr2	174854102	174854280	0.18	0.34085	0
chr2	175595400	175595575	0.18	-0.28759	0.00021
chr2	176967665	176968052	0.39	0.32984	0
chr2	179914864	179915033	0.17	0.32557	0
chr2	187463598	187463618	0.02	0.34912	0
chr2	190276864	190276928	0.06	0.30164	0.00021
chr2	190430929	190430998	0.07	0.36061	0
chr2	197124443	197124788	0.35	0.32864	0
chr2	197237686	197237764	0.08	0.31256	0
chr2	198540518	198540569	0.05	0.33916	0
chr2	200819070	200819113	0.04	-0.32755	0
chr2	201173446	201173803	0.36	0.3285	0
chr2	201725800	201726139	0.34	0.33786	0
chr2	201980467	201980504	0.04	-0.34484	0
chr2	202297914	202298223	0.31	0.33875	0
chr2	202561218	202561266	0.05	0.31187	0
chr2	202562775	202563587	0.81	0.36168	0
chr2	204102418	204102649	0.23	-0.32	0
chr2	208631081	208631684	0.6	-0.27504	0.00064
chr2	208635549	208635723	0.17	0.30803	0
chr2	208989248	208989382	0.13	0.31813	0
chr2	209224225	209224999	0.77	-0.31097	0
chr2	216616133	216616185	0.05	-0.3091	0
chr2	218231805	218231928	0.12	0.39962	0
chr2	218278052	218278111	0.06	0.37082	0
chr2	227659406	227659636	0.23	0.33337	0
chr2	229542809	229542880	0.07	-0.29422	0.00064
chr2	231833778	231834241	0.46	-0.33302	0
chr2	231988403	231988509	0.11	0.28396	0.00042
chr2	234296280	234297039	0.76	0.3093	0
chr2	238578491	238578727	0.24	0.32734	0
chr2	240143798	240144335	0.54	0.28933	0
chr2	240230587	240230892	0.31	0.32284	0
chr2	240866924	240867059	0.14	-0.35728	0
chr2	241095989	241096137	0.15	-0.30389	0
chr2	241458886	241460002	1.12	0.32619	0
chr2	242811830	242811864	0.03	-0.30291	0.00021

chr3	2933523	2933549	0.03	-0.2959	0.00064
chr3	3706853	3706868	0.02	-0.3019	0.00021
chr3	6784036	6784518	0.48	-0.30279	0.00021
chr3	10184319	10184877	0.56	-0.30329	0
chr3	12979917	12980202	0.29	0.30379	0.00021
chr3	15374534	15374586	0.05	-0.30004	0.00021
chr3	15823696	15823705	0.01	0.36647	0
chr3	25498670	25499105	0.44	0.30765	0
chr3	27761677	27762095	0.42	0.30273	0.00021
chr3	33701101	33701707	0.61	0.27817	0
chr3	37995561	37995838	0.28	0.30652	0
chr3	41280495	41280551	0.06	0.30605	0
chr3	41730160	41730177	0.02	0.31486	0
chr3	42108724	42109139	0.42	0.32551	0
chr3	45240278	45240318	0.04	0.3074	0
chr3	48216875	48216893	0.02	0.3419	0
chr3	53032818	53033167	0.35	-0.29622	0
chr3	57162032	57162039	0.01	0.41692	0
chr3	57881959	57882055	0.1	0.52643	0
chr3	64053561	64053663	0.1	0.32759	0
chr3	64193180	64193269	0.09	0.31564	0
chr3	64251663	64251919	0.26	0.33987	0
chr3	65387308	65387541	0.23	0.271	0.00085
chr3	70048832	70048926	0.09	-0.34578	0
chr3	71295335	71295390	0.06	0.31364	0
chr3	97982830	97983540	0.71	-0.2937	0
chr3	99904218	99904527	0.31	0.32714	0
chr3	101499090	101499503	0.41	0.29566	0.00064
chr3	106782411	106782589	0.18	0.34702	0
chr3	108180778	108180898	0.12	0.2995	0.00021
chr3	111456713	111456900	0.19	0.30901	0
chr3	114818612	114818700	0.09	0.37686	0
chr3	119379414	119379792	0.38	0.27272	0.00021
chr3	121311783	121312241	0.46	0.34616	0
chr3	121612944	121612956	0.01	0.373	0
chr3	123339334	123339918	0.58	0.37004	0
chr3	123409971	123410034	0.06	0.29268	0.00085
chr3	129276179	129276211	0.03	0.29601	0.00064
chr3	129407365	129407548	0.18	0.35061	0
chr3	129693385	129693586	0.2	0.32212	0
chr3	137361526	137361532	0.01	0.32417	0
chr3	141102599	141102964	0.37	0.35651	0
chr3	141150514	141151009	0.5	0.29456	0.00064
chr3	143033370	143033578	0.21	0.31192	0
chr3	147108843	147108916	0.07	0.29381	0.00064
chr3	147141219	147141588	0.37	0.31119	0
chr3	156260351	156260776	0.43	0.43661	0

chr3	156266748	156266974	0.23	0.3128	0
chr3	156795456	156795488	0.03	0.33421	0
chr3	157260593	157260627	0.03	0.36576	0
chr3	157812018	157812226	0.21	0.30084	0.00021
chr3	157813327	157813609	0.28	0.30086	0
chr3	160120464	160120481	0.02	-0.32578	0
chr3	171024571	171024841	0.27	0.29921	0
chr3	171561060	171561201	0.14	0.30895	0
chr3	177548098	177548306	0.21	0.38373	0
chr3	185271312	185271385	0.07	-0.42739	0
chr3	190105848	190105986	0.14	-0.48037	0
chr3	195870009	195870249	0.24	-0.30854	0
chr3	196065318	196065357	0.04	-0.27768	0.00042
chr3	196065506	196065569	0.06	-0.33814	0
chr3	197663873	197664106	0.23	-0.29702	0.00042
chr4	379900	380157	0.26	0.29883	0.00021
chr4	839528	839646	0.12	0.33602	0
chr4	1205359	1205447	0.09	0.31635	0
chr4	1411578	1411820	0.24	-0.30176	0.00021
chr4	2627014	2627194	0.18	0.38029	0
chr4	3306677	3306705	0.03	0.31176	0
chr4	4679368	4679628	0.26	0.30999	0
chr4	6537163	6537220	0.06	-0.32105	0
chr4	8321521	8321942	0.42	0.46369	0
chr4	12539867	12539883	0.02	-0.34842	0
chr4	15429515	15429795	0.28	0.31605	0
chr4	25881847	25881930	0.08	0.3097	0
chr4	37887255	37887467	0.21	0.35501	0
chr4	44018777	44018877	0.1	-0.29989	0.00021
chr4	46534014	46534428	0.41	-0.31893	0
chr4	54765826	54766004	0.18	0.31705	0
chr4	56685845	56685972	0.13	0.3218	0
chr4	57524758	57524770	0.01	0.30437	0.00021
chr4	57776726	57777244	0.52	-0.2897	0
chr4	57985198	57985260	0.06	0.29317	0.00085
chr4	71587640	71587904	0.26	0.29601	0
chr4	81185022	81185228	0.21	0.297	0.00042
chr4	83674996	83675797	0.8	0.35631	0
chr4	84071170	84071553	0.38	0.37057	0
chr4	88449925	88451496	1.57	0.43941	0
chr4	89152212	89152554	0.34	0.33789	0
chr4	95127681	95127703	0.02	-0.31927	0
chr4	100573753	100573850	0.1	-0.31546	0
chr4	100574455	100574653	0.2	-0.34064	0
chr4	100789493	100789506	0.01	0.33522	0
chr4	114213574	114213914	0.34	0.31868	0
chr4	114214093	114214449	0.36	0.33758	0

chr4	124570965	124571409	0.44	0.39478	0
chr4	140715901	140715994	0.09	0.31118	0
chr4	140808454	140808552	0.1	0.41652	0
chr4	148750768	148750770	0	0.3501	0
chr4	151505084	151505192	0.11	0.2992	0.00021
chr4	152097442	152097698	0.26	0.33535	0
chr4	152580806	152580863	0.06	0.34573	0
chr4	159094213	159094277	0.06	0.37122	0
chr4	165788672	165788674	0	0.33038	0
chr4	165898967	165899134	0.17	0.29977	0.00021
chr4	185118189	185118314	0.13	0.3348	0
chr4	187017924	187018050	0.13	0.32687	0
chr4	189064700	189064756	0.06	-0.31024	0
chr5	1193503	1193724	0.22	-0.30012	0.00021
chr5	1752668	1752864	0.2	-0.31019	0
chr5	2241205	2241248	0.04	-0.32322	0
chr5	2258129	2258598	0.47	-0.31206	0
chr5	2276656	2276682	0.03	-0.32723	0
chr5	2291750	2291952	0.2	-0.36836	0
chr5	2335681	2335723	0.04	-0.30887	0
chr5	2338616	2339100	0.48	-0.31225	0
chr5	2515177	2515396	0.22	-0.3034	0.00021
chr5	2645802	2645862	0.06	-0.39421	0
chr5	2866388	2866465	0.08	-0.32953	0
chr5	2964805	2965087	0.28	-0.3242	0
chr5	3339474	3339640	0.17	-0.29642	0
chr5	3822924	3823510	0.59	-0.33678	0
chr5	3932193	3932233	0.04	-0.31275	0
chr5	3961212	3962083	0.87	-0.32398	0
chr5	4851084	4851261	0.18	-0.29591	0.00064
chr5	4971058	4971641	0.58	-0.29048	0
chr5	5025803	5025848	0.05	-0.34934	0
chr5	5034397	5034730	0.33	-0.31485	0
chr5	5079059	5079159	0.1	-0.30584	0
chr5	5568539	5568588	0.05	-0.29752	0.00021
chr5	6030048	6030096	0.05	-0.31012	0
chr5	6411758	6411956	0.2	-0.2878	0.00021
chr5	14183373	14183872	0.5	0.35602	0
chr5	32445429	32445814	0.39	-0.29503	0
chr5	32584561	32584992	0.43	-0.31139	0
chr5	34504045	34504088	0.04	0.37532	0
chr5	36239673	36239818	0.15	0.30042	0.00021
chr5	40909520	40909532	0.01	0.30834	0
chr5	42953543	42953624	0.08	0.28876	0
chr5	43033934	43034043	0.11	0.32297	0
chr5	43037519	43037666	0.15	0.31216	0
chr5	44575014	44575156	0.14	-0.31752	0

chr5	50673033	50674015	0.98	0.30794	0
chr5	54518745	54519023	0.28	0.30258	0
chr5	55408419	55408595	0.18	-0.36923	0
chr5	55594154	55594466	0.31	0.30292	0.00021
chr5	58652891	58652948	0.06	0.30358	0
chr5	58653738	58654193	0.46	0.31211	0
chr5	59481426	59481615	0.19	0.28216	0.00042
chr5	60728124	60728164	0.04	0.33631	0
chr5	65105878	65106158	0.28	0.38665	0
chr5	66253919	66254876	0.96	0.30858	0
chr5	66445526	66445601	0.08	0.33527	0
chr5	67483109	67483245	0.14	0.34805	0
chr5	67588130	67588186	0.06	0.29945	0.00021
chr5	68326727	68326758	0.03	0.37557	0
chr5	74912210	74912492	0.28	0.31238	0
chr5	75903397	75903700	0.3	0.38045	0
chr5	76923988	76924190	0.2	0.29338	0.00085
chr5	76932016	76932062	0.05	0.29522	0
chr5	77253544	77253555	0.01	0.31295	0
chr5	77955963	77956305	0.34	0.31835	0
chr5	78203311	78203576	0.27	-0.32785	0
chr5	78407552	78407562	0.01	0.29758	0.00021
chr5	78407678	78407683	0.01	0.3376	0
chr5	82262468	82262646	0.18	0.29748	0.00021
chr5	92923396	92923623	0.23	0.33967	0
chr5	115379533	115379954	0.42	0.29719	0.00042
chr5	124711839	124711972	0.13	0.30602	0
chr5	125706935	125707117	0.18	0.27165	0.00085
chr5	131516837	131516877	0.04	0.27735	0.00042
chr5	134735637	134735654	0.02	0.29724	0.00042
chr5	138440656	138441071	0.42	0.35923	0
chr5	140457553	140457609	0.06	0.29012	0
chr5	140501365	140501451	0.09	0.3064	0
chr5	140613859	140613998	0.14	0.2966	0
chr5	140619446	140619586	0.14	0.30437	0
chr5	140624888	140625059	0.17	0.28753	0
chr5	140723455	140723549	0.09	0.30987	0
chr5	140743575	140743780	0.21	0.30949	0
chr5	140772148	140772182	0.03	0.28877	0
chr5	140778342	140778424	0.08	0.31267	0
chr5	140782277	140782510	0.23	0.29651	0
chr5	140798095	140798188	0.09	0.33524	0
chr5	140799541	140799572	0.03	0.31133	0
chr5	140801286	140801482	0.2	0.31121	0
chr5	140805681	140805841	0.16	0.34329	0
chr5	140810051	140810433	0.38	0.28981	0
chr5	140810920	140811102	0.18	0.36933	0

chr5	140811312	140811642	0.33	0.34398	0
chr5	142159386	142159393	0.01	0.41247	0
chr5	142922486	142922506	0.02	0.32142	0
chr5	158139210	158139306	0.1	-0.32911	0
chr5	159596570	159597030	0.46	0.31486	0
chr5	163893881	163894003	0.12	-0.34014	0
chr5	164668488	164668958	0.47	-0.2631	0.00085
chr5	167242169	167242266	0.1	-0.344	0
chr5	171830914	171831028	0.11	0.31627	0
chr5	172447213	172447638	0.43	0.30073	0.00021
chr5	173318556	173318755	0.2	0.32755	0
chr5	175969350	175969383	0.03	-0.29475	0.00064
chr5	180047184	180047407	0.22	-0.31531	0
chr5	180591594	180591704	0.11	0.29305	0.00085
chr6	1385731	1386142	0.41	0.30507	0
chr6	1391758	1392624	0.87	0.29062	0
chr6	5132887	5133188	0.3	0.3079	0
chr6	6737080	6737631	0.55	0.27298	0.00085
chr6	10390811	10391412	0.6	0.34583	0
chr6	10393497	10393778	0.28	0.37505	0
chr6	10416373	10416531	0.16	0.27189	0.00021
chr6	10417087	10418121	1.03	0.27257	0
chr6	10422636	10422874	0.24	0.34371	0
chr6	10555682	10555881	0.2	0.32784	0
chr6	11607923	11607982	0.06	0.33285	0
chr6	11805824	11806115	0.29	-0.34553	0
chr6	13874574	13874679	0.11	-0.41019	0
chr6	15463247	15463343	0.1	0.37923	0
chr6	16337711	16337921	0.21	0.3017	0.00021
chr6	16352321	16352745	0.42	0.33974	0
chr6	16747686	16748160	0.47	0.41707	0
chr6	17289613	17290146	0.53	0.29138	0
chr6	17470162	17470599	0.44	0.31587	0
chr6	18824218	18824224	0.01	0.30075	0.00021
chr6	22056238	22056734	0.5	0.29446	0.00064
chr6	25650786	25651144	0.36	-0.29926	0.00021
chr6	26225246	26225407	0.16	0.30462	0
chr6	26226203	26226256	0.05	0.35531	0
chr6	27173633	27173991	0.36	0.32536	0
chr6	27730551	27730563	0.01	0.30283	0.00021
chr6	27832098	27832180	0.08	0.33344	0
chr6	28058724	28059208	0.48	0.33345	0
chr6	28226885	28227482	0.6	0.31662	0
chr6	28945322	28945341	0.02	0.29126	0
chr6	30130819	30131001	0.18	-0.35195	0
chr6	30420981	30421089	0.11	0.29441	0.00064
chr6	31734147	31734580	0.43	0.29511	0

chr6	31746808	31746814	0.01	-0.33121	0
chr6	31938773	31938847	0.07	-0.29357	0
chr6	32060681	32061084	0.4	0.2997	0
chr6	33173482	33173501	0.02	-0.30023	0
chr6	34482460	34482679	0.22	0.30737	0
chr6	34984836	34984930	0.09	0.33406	0
chr6	35479628	35479648	0.02	0.33164	0
chr6	38672050	38672343	0.29	0.34808	0
chr6	42145954	42146052	0.1	0.35387	0
chr6	42788563	42789053	0.49	0.32319	0
chr6	43082234	43082296	0.06	0.36877	0
chr6	43117933	43118059	0.13	0.31741	0
chr6	43478692	43478829	0.14	-0.27201	0.00085
chr6	47066614	47066683	0.07	0.36406	0
chr6	48842056	48842122	0.07	-0.32292	0
chr6	50480396	50480537	0.14	-0.30275	0.00021
chr6	50481291	50481652	0.36	-0.30695	0
chr6	76203642	76204172	0.53	0.27342	0.00021
chr6	76802339	76802592	0.25	-0.29536	0
chr6	90271380	90271989	0.61	0.29232	0
chr6	90272057	90272492	0.44	0.31643	0
chr6	92401486	92401545	0.06	-0.37791	0
chr6	100911709	100911746	0.04	0.28316	0.00042
chr6	100915395	100915805	0.41	0.29232	0
chr6	100916916	100916959	0.04	0.29494	0.00064
chr6	101846797	101846805	0.01	0.31344	0
chr6	105388153	105388191	0.04	0.29437	0.00064
chr6	105388668	105388731	0.06	0.31593	0
chr6	105400884	105400993	0.11	0.37056	0
chr6	108492653	108492769	0.12	0.28139	0.00042
chr6	108977419	108977456	0.04	0.29545	0.00064
chr6	109072250	109072674	0.42	-0.31768	0
chr6	109274429	109274464	0.04	0.32559	0
chr6	109778433	109778491	0.06	0.31893	0
chr6	111194645	111194786	0.14	-0.29428	0.00064
chr6	111688745	111689217	0.47	0.38203	0
chr6	111888540	111888567	0.03	0.3033	0.00021
chr6	111888652	111888683	0.03	0.29393	0
chr6	111920891	111920977	0.09	0.40711	0
chr6	111947606	111947780	0.17	0.29382	0.00064
chr6	111968372	111968560	0.19	0.3155	0
chr6	112572461	112572936	0.48	0.35709	0
chr6	134589516	134589589	0.07	-0.3526	0
chr6	135376695	135376777	0.08	-0.31714	0
chr6	138866882	138867125	0.24	0.32631	0
chr6	144223298	144223614	0.32	0.35977	0
chr6	144904205	144904306	0.1	0.31545	0

chr6	150023044	150023289	0.25	0.30251	0
chr6	151521252	151521637	0.39	0.32894	0
chr6	151937260	151937653	0.39	0.31969	0
chr6	152011103	152011415	0.31	0.30174	0
chr6	152702330	152702660	0.33	0.30679	0
chr6	152723267	152723414	0.15	-0.29529	0.00064
chr6	157161359	157161545	0.19	0.32538	0
chr6	157464871	157465138	0.27	0.30343	0.00021
chr6	159487527	159487544	0.02	0.33485	0
chr6	161678989	161679078	0.09	0.3251	0
chr6	166247032	166247348	0.32	-0.31335	0
chr6	168378673	168378810	0.14	-0.32657	0
chr6	168379043	168379113	0.07	-0.34393	0
chr6	169350747	169351479	0.73	0.28614	0
chr6	169399712	169399762	0.05	-0.2963	0
chr6	169740686	169740781	0.1	-0.34732	0
chr6	170361474	170361827	0.35	0.32186	0
chr7	543447	543509	0.06	0.31469	0
chr7	675530	676094	0.56	-0.34818	0
chr7	705435	705693	0.26	0.31868	0
chr7	810958	811786	0.83	0.39183	0
chr7	837297	837536	0.24	0.33159	0
chr7	881634	881651	0.02	0.29994	0.00021
chr7	1303351	1303448	0.1	-0.37472	0
chr7	1333612	1333912	0.3	-0.3001	0.00021
chr7	1437272	1437409	0.14	-0.31311	0
chr7	1718485	1718541	0.06	-0.28694	0.00021
chr7	1793077	1793191	0.11	-0.29065	0
chr7	2144559	2144579	0.02	0.3099	0
chr7	4746837	4747026	0.19	0.30355	0
chr7	4747216	4747261	0.05	0.31528	0
chr7	4848814	4848939	0.13	-0.33142	0
chr7	5711782	5712269	0.49	0.33518	0
chr7	12410026	12410033	0.01	0.31512	0
chr7	18678293	18678642	0.35	0.31243	0
chr7	23387365	23387575	0.21	-0.31703	0
chr7	27160520	27160960	0.44	0.29721	0
chr7	27181067	27181671	0.6	0.28632	0
chr7	29519323	29519656	0.33	0.28803	0
chr7	30810183	30810504	0.32	-0.34235	0
chr7	33196871	33197291	0.42	0.30177	0.00021
chr7	33767199	33767428	0.23	-0.29554	0.00064
chr7	34361111	34361199	0.09	-0.35642	0
chr7	42237160	42237350	0.19	-0.37753	0
chr7	43288411	43289038	0.63	-0.32047	0
chr7	43652291	43652643	0.35	0.35355	0
chr7	47431773	47432311	0.54	0.29572	0

chr7	47475566	47475653	0.09	0.30524	0.00021
chr7	53103789	53104336	0.55	-0.27385	0.00064
chr7	56297564	56297579	0.02	0.31509	0
chr7	65579017	65579080	0.06	-0.28827	0.00021
chr7	66398301	66398313	0.01	0.29538	0.00064
chr7	71868406	71868494	0.09	-0.3799	0
chr7	75596992	75597100	0.11	0.28116	0.00042
chr7	80231483	80231599	0.12	0.29111	0
chr7	87941609	87941769	0.16	0.30028	0.00021
chr7	95115163	95115232	0.07	0.32211	0
chr7	96622311	96622709	0.4	0.28275	0.00042
chr7	97952898	97953106	0.21	0.31376	0
chr7	99229440	99229650	0.21	0.33567	0
chr7	100881113	100881325	0.21	0.29538	0
chr7	101518619	101518899	0.28	0.474	0
chr7	101579929	101580127	0.2	0.36622	0
chr7	101799198	101799218	0.02	0.35202	0
chr7	102574105	102574445	0.34	0.30273	0
chr7	102790406	102790498	0.09	-0.29612	0.00064
chr7	107235907	107236140	0.23	0.34274	0
chr7	110731116	110731201	0.09	0.3076	0
chr7	116654858	116654903	0.05	0.34597	0
chr7	117119195	117119637	0.44	0.29825	0
chr7	129868685	129868786	0.1	0.3176	0
chr7	130125511	130125756	0.25	-0.33787	0
chr7	130125932	130125976	0.04	-0.28837	0.00021
chr7	130646051	130646078	0.03	-0.36311	0
chr7	134571320	134571494	0.17	0.37587	0
chr7	137659854	137660322	0.47	0.33405	0
chr7	137749927	137750050	0.12	0.3614	0
chr7	138312290	138312324	0.03	0.31943	0
chr7	143582499	143582511	0.01	0.38368	0
chr7	143582630	143582650	0.02	0.30923	0
chr7	149112318	149112402	0.08	0.30645	0
chr7	150686728	150686735	0.01	0.32485	0
chr7	151565595	151565731	0.14	0.27304	0.00021
chr7	151840010	151840170	0.16	0.32283	0
chr7	154401472	154401618	0.15	-0.31136	0
chr7	154542235	154542313	0.08	-0.29896	0.00021
chr7	154867682	154867763	0.08	-0.30702	0
chr7	155581411	155581623	0.21	-0.33498	0
chr7	155744340	155744544	0.2	-0.40551	0
chr7	156943639	156943877	0.24	0.33247	0
chr7	157261163	157261603	0.44	-0.2935	0.00064
chr7	157280713	157281332	0.62	-0.32736	0
chr7	157293538	157293544	0.01	-0.44856	0
chr7	157294107	157294502	0.4	-0.31741	0

chr7	157320369	157320516	0.15	-0.27921	0.00042
chr7	157332818	157333010	0.19	-0.32093	0
chr7	157334162	157334648	0.49	-0.30356	0.00021
chr7	157340390	157340554	0.16	-0.32622	0
chr7	157345566	157345659	0.09	-0.33967	0
chr7	157345827	157347375	1.55	-0.30321	0
chr7	157351620	157351708	0.09	-0.36094	0
chr7	157352978	157353191	0.21	-0.295	0
chr7	157357887	157357947	0.06	-0.3244	0
chr7	157367142	157367871	0.73	-0.31268	0
chr7	157391030	157391297	0.27	-0.27534	0.00064
chr7	157402529	157402637	0.11	-0.31118	0
chr7	157408632	157408770	0.14	-0.32194	0
chr7	157411039	157411142	0.1	-0.30248	0.00021
chr7	157412886	157413057	0.17	-0.30435	0.00021
chr7	157423165	157423211	0.05	-0.35672	0
chr7	157435200	157435297	0.1	-0.30743	0
chr7	157438537	157438616	0.08	-0.29615	0.00064
chr7	157460477	157460657	0.18	-0.30831	0
chr7	157467966	157468132	0.17	-0.2974	0
chr7	157502732	157502966	0.23	-0.30948	0
chr7	157514730	157514822	0.09	-0.31024	0
chr7	157523356	157523541	0.19	-0.29531	0
chr7	157534363	157534758	0.4	-0.30213	0.00021
chr7	157568273	157568392	0.12	-0.30779	0
chr7	157579745	157579880	0.14	-0.36406	0
chr7	157584081	157584183	0.1	-0.33008	0
chr7	157605569	157605684	0.12	-0.39039	0
chr7	157620445	157620685	0.24	-0.36166	0
chr7	157622032	157622138	0.11	-0.33878	0
chr7	157624788	157624958	0.17	-0.28637	0.00021
chr7	157630174	157630250	0.08	-0.33351	0
chr7	157691541	157691591	0.05	-0.33692	0
chr7	157695026	157695230	0.2	-0.30166	0.00021
chr7	157695428	157695747	0.32	-0.30717	0
chr7	157738245	157738287	0.04	-0.29754	0.00021
chr7	157744316	157744347	0.03	-0.42483	0
chr7	157794069	157794688	0.62	-0.33913	0
chr7	157854693	157854772	0.08	-0.2974	0
chr7	157867291	157867695	0.4	-0.26586	0.00064
chr7	157916077	157916580	0.5	-0.28745	0
chr7	157919649	157919982	0.33	-0.28464	0.00042
chr7	157932168	157933354	1.19	-0.30598	0
chr7	157935557	157935968	0.41	-0.31764	0
chr7	157950418	157950633	0.22	-0.30919	0
chr7	157951471	157951799	0.33	-0.30328	0.00021
chr7	157953501	157953935	0.43	-0.30218	0.00021

chr7	157955692	157956042	0.35	-0.3515	0
chr7	157957646	157958261	0.62	-0.35761	0
chr7	157958671	157958806	0.14	-0.29235	0
chr7	157968629	157968683	0.05	-0.32499	0
chr7	157981337	157981849	0.51	-0.28273	0.00042
chr7	157985113	157985171	0.06	-0.32088	0
chr7	158037729	158038118	0.39	-0.27099	0.00021
chr7	158045980	158046358	0.38	-0.31698	0
chr7	158050413	158050618	0.21	-0.29894	0.00021
chr7	158059396	158059704	0.31	-0.30106	0.00021
chr7	158072687	158073079	0.39	-0.33733	0
chr7	158093250	158093277	0.03	-0.30389	0.00021
chr7	158107284	158107723	0.44	-0.27754	0.00042
chr7	158279818	158279846	0.03	-0.31753	0
chr7	158349388	158349441	0.05	-0.31283	0
chr7	158354692	158355003	0.31	-0.32433	0
chr7	158575681	158575934	0.25	0.28067	0.00042
chr8	1051703	1051814	0.11	-0.37271	0
chr8	1054487	1054717	0.23	-0.35182	0
chr8	1136200	1136444	0.24	-0.26824	0.00042
chr8	1311063	1311576	0.51	-0.27533	0.00021
chr8	1339049	1340584	1.54	-0.30193	0
chr8	1443368	1443908	0.54	-0.27216	0.00085
chr8	1448361	1449634	1.27	-0.28978	0
chr8	1497878	1498266	0.39	-0.31248	0
chr8	1906312	1906338	0.03	0.31	0
chr8	1971471	1971479	0.01	-0.30709	0
chr8	2024193	2024249	0.06	-0.30901	0
chr8	2088209	2088595	0.39	-0.27293	0.00085
chr8	2419822	2420061	0.24	-0.34557	0
chr8	3047536	3047756	0.22	-0.35485	0
chr8	3245076	3245147	0.07	-0.3338	0
chr8	3565626	3566118	0.49	-0.33743	0
chr8	3855415	3855502	0.09	-0.2929	0.00085
chr8	4987083	4987358	0.28	-0.31027	0
chr8	6323131	6323299	0.17	0.41137	0
chr8	6420726	6420770	0.04	0.31706	0
chr8	8748536	8748792	0.26	-0.36029	0
chr8	9105451	9105620	0.17	0.33771	0
chr8	13706623	13707244	0.62	-0.27215	0.00085
chr8	17258080	17258246	0.17	0.34789	0
chr8	17554904	17555302	0.4	0.27301	0.00064
chr8	22925443	22925455	0.01	-0.30433	0.00021
chr8	27466501	27466508	0.01	0.38097	0
chr8	28912915	28913104	0.19	0.31255	0
chr8	29356671	29357029	0.36	-0.3371	0
chr8	29732519	29732547	0.03	0.32401	0

chr8	29732579	29732714	0.14	0.30496	0
chr8	30244710	30245118	0.41	0.34449	0
chr8	30401028	30401046	0.02	0.37052	0
chr8	30420852	30420906	0.05	0.34819	0
chr8	35581557	35581595	0.04	-0.31681	0
chr8	35718561	35718698	0.14	-0.32646	0
chr8	35953501	35953736	0.24	-0.33381	0
chr8	37457295	37457573	0.28	0.30949	0
chr8	38562303	38562556	0.25	0.3206	0
chr8	38627889	38628418	0.53	0.30345	0
chr8	42066048	42066228	0.18	0.33291	0
chr8	42103037	42103068	0.03	0.30666	0
chr8	42125496	42125522	0.03	0.44201	0
chr8	42358481	42359099	0.62	0.41008	0
chr8	48044616	48044856	0.24	-0.40628	0
chr8	48920450	48920460	0.01	-0.31941	0
chr8	50080767	50081162	0.4	-0.2788	0.00042
chr8	50241884	50241940	0.06	-0.31093	0
chr8	50424882	50425219	0.34	-0.31343	0
chr8	50653870	50653901	0.03	-0.34892	0
chr8	52721802	52722237	0.44	0.30116	0
chr8	59058660	59059235	0.58	-0.31338	0
chr8	65525631	65525669	0.04	-0.30304	0.00021
chr8	67472194	67472493	0.3	0.43866	0
chr8	67975874	67975895	0.02	-0.32985	0
chr8	67981092	67981112	0.02	0.31375	0
chr8	70246362	70246492	0.13	-0.30122	0.00021
chr8	71069942	71070339	0.4	0.35861	0
chr8	80836065	80836322	0.26	0.31957	0
chr8	82530924	82530967	0.04	0.33462	0
chr8	83073052	83073193	0.14	-0.31994	0
chr8	86568078	86568163	0.09	-0.29675	0
chr8	91952744	91953125	0.38	0.3179	0
chr8	96393868	96394047	0.18	0.36904	0
chr8	99951151	99951730	0.58	0.3084	0
chr8	99955439	99955610	0.17	0.30191	0
chr8	99959430	99959602	0.17	0.29391	0
chr8	99961122	99962347	1.23	0.36794	0
chr8	103395266	103395625	0.36	0.35358	0
chr8	103741780	103742123	0.34	0.31245	0
chr8	109800338	109800379	0.04	-0.30007	0.00021
chr8	110380981	110381021	0.04	0.44953	0
chr8	110615382	110615865	0.48	0.29293	0.00085
chr8	121208577	121208681	0.1	0.34471	0
chr8	121825470	121825702	0.23	-0.30354	0.00021
chr8	124526525	124526866	0.34	0.34178	0
chr8	126082836	126083119	0.28	0.30218	0.00021

chr8	128808017	128808262	0.25	-0.31715	0
chr8	129061191	129061382	0.19	0.3801	0
chr8	138493473	138493933	0.46	-0.33263	0
chr8	139074557	139074614	0.06	-0.27455	0.00064
chr8	140119546	140120087	0.54	-0.30987	0
chr8	140630155	140630180	0.03	-0.2988	0.00021
chr8	143623372	143623661	0.29	-0.28625	0.00021
chr8	143694305	143694620	0.32	-0.31818	0
chr8	144639901	144640043	0.14	-0.30302	0.00021
chr8	144797929	144797967	0.04	-0.28159	0.00042
chr9	841067	841270	0.2	0.30487	0.00021
chr9	6215327	6215640	0.31	0.28662	0.00021
chr9	7147104	7147265	0.16	0.34131	0
chr9	14180661	14180824	0.16	0.39989	0
chr9	14563979	14564157	0.18	0.27985	0.00042
chr9	21966564	21967469	0.91	0.33614	0
chr9	35538922	35539509	0.59	0.26846	0.00042
chr9	71440805	71440828	0.02	0.29501	0.00064
chr9	84228185	84228672	0.49	0.3089	0
chr9	85019920	85020050	0.13	0.38185	0
chr9	89422493	89422603	0.11	0.40649	0
chr9	90452294	90452467	0.17	0.28924	0
chr9	95187808	95188039	0.23	0.34709	0
chr9	95244844	95244874	0.03	0.33773	0
chr9	95969598	95970064	0.47	0.29502	0.00064
chr9	97818542	97818968	0.43	0.33038	0
chr9	100617999	100618115	0.12	0.3232	0
chr9	100850334	100850337	0	0.37378	0
chr9	109684300	109684453	0.15	0.30498	0.00021
chr9	117150236	117150495	0.26	0.30442	0
chr9	124979184	124979356	0.17	0.35258	0
chr9	125795488	125795935	0.45	0.29835	0
chr9	127019325	127019707	0.38	-0.28182	0.00021
chr9	128235091	128235430	0.34	0.36479	0
chr9	129972490	129972678	0.19	0.30099	0.00021
chr9	130853572	130854132	0.56	0.30747	0
chr9	131679477	131679780	0.3	0.32656	0
chr9	133567787	133567903	0.12	0.32738	0
chr9	138289410	138289504	0.09	-0.31035	0
chr9	140400563	140400693	0.13	-0.39224	0
chr10	616959	617105	0.15	0.33241	0
chr10	695844	695900	0.06	0.37141	0
chr10	1195844	1195916	0.07	0.31025	0
chr10	1715848	1715879	0.03	-0.29246	0.00085
chr10	1850312	1850372	0.06	-0.32857	0
chr10	2125396	2125911	0.52	-0.28197	0.00042
chr10	2541463	2541820	0.36	-0.30926	0

chr10	2699291	2699357	0.07	-0.34277	0
chr10	3329765	3329966	0.2	-0.35541	0
chr10	3805252	3805441	0.19	-0.39363	0
chr10	5060432	5060566	0.13	0.33507	0
chr10	7088654	7088771	0.12	-0.30237	0.00021
chr10	7215201	7215341	0.14	-0.31991	0
chr10	11047435	11047586	0.15	-0.30268	0.00021
chr10	11615774	11615853	0.08	0.29212	0.00085
chr10	11799636	11799667	0.03	0.35932	0
chr10	16250844	16251115	0.27	-0.32417	0
chr10	16319997	16320001	0	-0.34342	0
chr10	16320541	16320652	0.11	-0.32215	0
chr10	18239805	18240316	0.51	-0.28376	0.00042
chr10	22622459	22622793	0.33	0.29805	0.00021
chr10	22843607	22843938	0.33	0.30014	0.00021
chr10	24738227	24738322	0.1	0.29708	0.00042
chr10	29980953	29981216	0.26	0.31643	0
chr10	30020120	30020140	0.02	0.37799	0
chr10	34313874	34313965	0.09	0.29745	0.00021
chr10	34391256	34391668	0.41	0.33184	0
chr10	45923083	45923705	0.62	0.313	0
chr10	50143375	50143509	0.13	0.31422	0
chr10	60086738	60087111	0.37	0.34518	0
chr10	64679758	64679766	0.01	-0.30647	0
chr10	64967241	64967610	0.37	0.33205	0
chr10	65442289	65442359	0.07	0.30985	0
chr10	70322442	70322874	0.43	0.30185	0.00021
chr10	72454006	72454026	0.02	0.30647	0
chr10	73272256	73272300	0.04	-0.2987	0.00021
chr10	73746656	73746786	0.13	0.33101	0
chr10	79003155	79003508	0.35	0.3174	0
chr10	93805441	93805682	0.24	0.30994	0
chr10	94452311	94452554	0.24	0.30671	0
chr10	98129751	98130310	0.56	-0.40185	0
chr10	99393873	99393886	0.01	-0.323	0
chr10	102496500	102496915	0.42	0.32507	0
chr10	102642655	102642752	0.1	0.32814	0
chr10	103698992	103699237	0.25	0.33738	0
chr10	104905387	104905806	0.42	0.36249	0
chr10	107292939	107292956	0.02	-0.30326	0.00021
chr10	109135976	109136178	0.2	-0.30051	0.00021
chr10	112723024	112723296	0.27	0.37043	0
chr10	116335842	116336057	0.22	0.33732	0
chr10	116367648	116367729	0.08	0.3293	0
chr10	119291766	119292062	0.3	0.32089	0
chr10	119292300	119292549	0.25	0.3047	0
chr10	119293341	119293550	0.21	0.29061	0

chr10	120115553	120115780	0.23	0.29626	0.00064
chr10	120429062	120429205	0.14	0.29269	0
chr10	121300857	121301041	0.18	-0.29804	0.00021
chr10	124459311	124459513	0.2	-0.28742	0.00021
chr10	126392111	126392429	0.32	0.33157	0
chr10	126635660	126636047	0.39	0.33866	0
chr10	126782397	126782576	0.18	0.31502	0
chr10	128607975	128608036	0.06	0.34435	0
chr10	130124378	130124853	0.48	-0.29408	0
chr10	130185305	130185387	0.08	-0.32493	0
chr10	130201069	130201251	0.18	-0.29206	0.00085
chr10	130422176	130422194	0.02	-0.30635	0
chr10	130832268	130832393	0.13	-0.33672	0
chr10	131843725	131844548	0.82	-0.29083	0
chr10	132461246	132461281	0.04	-0.30925	0
chr10	132583721	132584219	0.5	-0.31824	0
chr10	132882986	132883127	0.14	-0.34523	0
chr10	132886634	132886739	0.11	-0.27777	0.00042
chr10	132891319	132891371	0.05	-0.31701	0
chr10	132891754	132892187	0.43	-0.33938	0
chr10	132897099	132897160	0.06	-0.2985	0.00021
chr10	132999672	132999707	0.04	-0.30703	0
chr10	133036065	133036108	0.04	-0.34065	0
chr10	133055449	133055651	0.2	-0.31904	0
chr10	133058901	133058988	0.09	-0.3009	0.00021
chr10	133208644	133208984	0.34	-0.31429	0
chr10	133317423	133318287	0.86	-0.31571	0
chr10	133508385	133508442	0.06	-0.33827	0
chr10	133598589	133598669	0.08	-0.3737	0
chr10	134143489	134143535	0.05	-0.35662	0
chr10	134144355	134144401	0.05	-0.41011	0
chr10	134334241	134334283	0.04	0.29443	0.00064
chr10	134623278	134624055	0.78	-0.30101	0
chr10	134647690	134648115	0.43	-0.30255	0
chr10	134843768	134843775	0.01	-0.33768	0
chr10	134875720	134875916	0.2	-0.36493	0
chr10	134977297	134978147	0.85	-0.31842	0
chr10	134994424	134994945	0.52	-0.30108	0
chr10	134996789	134996895	0.11	-0.36726	0
chr10	135002718	135002976	0.26	-0.29733	0
chr10	135012454	135012575	0.12	-0.32647	0
chr10	135017785	135018992	1.21	-0.35897	0
chr10	135020931	135021100	0.17	-0.29938	0
chr10	135030513	135030554	0.04	-0.29316	0.00085
chr10	135039948	135040250	0.3	-0.36168	0
chr10	135123006	135123337	0.33	-0.32563	0
chr10	135153937	135153972	0.04	-0.35584	0

chr11	460587	460914	0.33	-0.28344	0.00042
chr11	1321929	1321954	0.03	0.33748	0
chr11	2705153	2705357	0.2	0.3005	0.00021
chr11	4928572	4928579	0.01	-0.31662	0
chr11	5129538	5129836	0.3	-0.29131	0
chr11	5264613	5265215	0.6	-0.29536	0
chr11	5346163	5346556	0.39	-0.29703	0
chr11	5372087	5372944	0.86	-0.30668	0
chr11	5951048	5951433	0.39	-0.36123	0
chr11	6518438	6518792	0.35	0.31027	0
chr11	8653170	8653329	0.16	0.30482	0.00021
chr11	8705790	8705801	0.01	-0.31484	0
chr11	10750870	10750890	0.02	0.43749	0
chr11	11994913	11995462	0.55	0.28402	0.00042
chr11	12180609	12180973	0.36	0.3851	0
chr11	12182350	12182377	0.03	0.29185	0.00085
chr11	12875566	12875633	0.07	0.35405	0
chr11	13983009	13983851	0.84	-0.30637	0
chr11	31818474	31818791	0.32	0.29299	0.00085
chr11	35019666	35019673	0.01	0.34068	0
chr11	35704400	35704643	0.24	0.31999	0
chr11	35834513	35834595	0.08	0.34847	0
chr11	36184661	36184683	0.02	0.35358	0
chr11	43945505	43945562	0.06	0.30864	0
chr11	45070809	45070822	0.01	0.3364	0
chr11	47510560	47510670	0.11	0.29204	0.00085
chr11	55587090	55587104	0.01	-0.30713	0
chr11	55796741	55797190	0.45	-0.29856	0.00021
chr11	55798206	55798258	0.05	-0.30901	0
chr11	56141829	56141860	0.03	-0.30587	0
chr11	56466578	56466770	0.19	-0.28	0.00042
chr11	56510971	56511248	0.28	-0.3078	0
chr11	59823508	59823993	0.49	-0.30888	0
chr11	59929200	59929511	0.31	-0.29786	0.00021
chr11	60282398	60282420	0.02	-0.29744	0.00021
chr11	60282712	60282742	0.03	-0.30208	0.00021
chr11	61159047	61159083	0.04	-0.31233	0
chr11	62303750	62304165	0.42	0.3343	0
chr11	64097027	64097101	0.07	0.29227	0
chr11	64322890	64323247	0.36	0.33019	0
chr11	64863232	64863373	0.14	0.30388	0.00021
chr11	64871778	64871888	0.11	0.41911	0
chr11	68782049	68782202	0.15	0.30113	0
chr11	68853915	68853957	0.04	0.35014	0
chr11	69706285	69706527	0.24	-0.3171	0
chr11	69706831	69706901	0.07	-0.29651	0.00042
chr11	69707303	69707384	0.08	-0.38261	0

chr11	69949774	69950073	0.3	0.30387	0.00021
chr11	70210616	70210622	0.01	0.34191	0
chr11	70289549	70289583	0.03	-0.28669	0.00021
chr11	72019825	72019901	0.08	0.3534	0
chr11	76474735	76474788	0.05	0.31244	0
chr11	86105255	86105321	0.07	0.30222	0.00021
chr11	102702285	102702414	0.13	0.29191	0.00085
chr11	105590209	105590390	0.18	-0.34369	0
chr11	113244828	113245069	0.24	0.32022	0
chr11	118657127	118657363	0.24	0.3076	0
chr11	119524154	119524243	0.09	-0.31703	0
chr11	120618705	120618722	0.02	0.29563	0.00064
chr11	120998006	120998811	0.81	-0.33062	0
chr11	121208950	121208989	0.04	0.40174	0
chr11	121365540	121365727	0.19	0.29183	0.00085
chr11	122655631	122655638	0.01	0.41752	0
chr11	123885349	123885695	0.35	-0.32275	0
chr11	124506014	124506266	0.25	0.32314	0
chr11	125801456	125801519	0.06	0.35433	0
chr11	129145940	129145951	0.01	0.32753	0
chr11	131778850	131779255	0.41	-0.35213	0
chr11	131799287	131799701	0.41	-0.28823	0
chr11	132016192	132016295	0.1	-0.33954	0
chr11	132080258	132080630	0.37	-0.35354	0
chr11	132107419	132107534	0.12	-0.3718	0
chr11	132177605	132177693	0.09	-0.28308	0.00042
chr11	132394504	132394991	0.49	-0.32609	0
chr11	132526947	132527438	0.49	-0.33377	0
chr11	134783448	134783504	0.06	-0.30586	0
chr12	272635	272745	0.11	-0.32286	0
chr12	2082146	2082218	0.07	0.35185	0
chr12	2968001	2968215	0.21	0.31883	0
chr12	3011514	3011741	0.23	0.35525	0
chr12	3361896	3361996	0.1	0.29734	0.00021
chr12	6501933	6501960	0.03	0.31066	0
chr12	6665335	6665424	0.09	0.28593	0.00021
chr12	7858592	7858598	0.01	-0.29359	0.00064
chr12	7942353	7942817	0.46	0.35483	0
chr12	8025394	8025435	0.04	0.3192	0
chr12	8025646	8025756	0.11	0.31074	0
chr12	9478612	9478906	0.29	0.29643	0
chr12	11877740	11878017	0.28	0.30578	0
chr12	12659177	12659685	0.51	0.31062	0
chr12	13132607	13132661	0.05	0.29406	0.00064
chr12	15782193	15782294	0.1	0.31403	0
chr12	16034055	16034100	0.05	-0.30947	0
chr12	16762789	16762843	0.05	0.30452	0

chr12	16942296	16942300	0	0.33175	0
chr12	18890833	18890872	0.04	-0.31701	0
chr12	19358223	19358289	0.07	0.39727	0
chr12	26038809	26038924	0.12	-0.33191	0
chr12	32825282	32825307	0.03	0.36125	0
chr12	34474362	34474637	0.28	-0.31912	0
chr12	34488729	34488830	0.1	-0.3734	0
chr12	34494825	34494852	0.03	-0.37965	0
chr12	42628974	42629138	0.16	0.54352	0
chr12	46776389	46776425	0.04	-0.2929	0.00085
chr12	46855182	46855622	0.44	0.32578	0
chr12	50528616	50528754	0.14	0.3911	0
chr12	51421225	51421367	0.14	-0.34544	0
chr12	52652377	52652462	0.09	0.29368	0.00064
chr12	53995468	53995504	0.04	0.33272	0
chr12	57735981	57736363	0.38	0.30699	0
chr12	64687265	64687325	0.06	-0.34168	0
chr12	66220754	66220810	0.06	-0.39799	0
chr12	69236712	69236811	0.1	0.32827	0
chr12	69752753	69753119	0.37	-0.2954	0
chr12	89033255	89033280	0.03	0.30005	0.00021
chr12	91572191	91572199	0.01	0.30784	0
chr12	93396260	93396759	0.5	0.3247	0
chr12	94131844	94131957	0.11	0.37833	0
chr12	94208017	94208276	0.26	0.39482	0
chr12	95945384	95945610	0.23	0.2927	0
chr12	96350720	96350795	0.08	0.33857	0
chr12	96633384	96633723	0.34	0.344	0
chr12	103633300	103633338	0.04	-0.3181	0
chr12	104194358	104194370	0.01	0.32204	0
chr12	105065028	105065386	0.36	-0.34545	0
chr12	105626937	105627190	0.25	0.306	0
chr12	109896495	109897234	0.74	0.29827	0
chr12	110172208	110172346	0.14	0.30208	0
chr12	111926384	111926423	0.04	0.31357	0
chr12	113916609	113916664	0.06	0.36477	0
chr12	114513507	114513561	0.05	-0.31796	0
chr12	118627943	118628449	0.51	0.31582	0
chr12	119086607	119086637	0.03	-0.32737	0
chr12	119213093	119213160	0.07	-0.35121	0
chr12	119213900	119214021	0.12	-0.38193	0
chr12	119346888	119347315	0.43	-0.29845	0.00021
chr12	120242394	120242513	0.12	0.28002	0.00042
chr12	120687694	120688060	0.37	0.31969	0
chr12	122764418	122764545	0.13	0.32327	0
chr12	123930117	123930124	0.01	0.33883	0
chr12	124811985	124812154	0.17	0.46561	0

chr12	126926911	126926925	0.01	-0.29364	0.00064
chr12	128245593	128245658	0.07	-0.27563	0.00042
chr12	128602715	128602780	0.07	-0.30894	0
chr12	129594978	129595394	0.42	-0.33357	0
chr12	130183692	130184190	0.5	-0.31238	0
chr12	130184740	130185259	0.52	-0.31199	0
chr12	130333815	130334009	0.19	-0.35043	0
chr12	130335340	130335752	0.41	-0.33569	0
chr12	130337265	130337671	0.41	-0.32256	0
chr12	130420637	130420666	0.03	-0.31985	0
chr12	130958711	130958790	0.08	-0.29879	0.00021
chr12	131417943	131418217	0.27	-0.31627	0
chr12	132677496	132677645	0.15	-0.30021	0.00021
chr12	132863782	132863983	0.2	-0.36329	0
chr12	132880790	132881276	0.49	-0.41172	0
chr12	132983481	132983814	0.33	-0.308	0
chr12	133345285	133345322	0.04	0.30293	0.00021
chr13	25085301	25085669	0.37	-0.43225	0
chr13	25668392	25668714	0.32	-0.32648	0
chr13	28545214	28545295	0.08	0.35827	0
chr13	30951587	30952016	0.43	0.43204	0
chr13	30963379	30963558	0.18	0.30837	0
chr13	31456352	31456604	0.25	0.33342	0
chr13	39989296	39989389	0.09	0.29669	0.00042
chr13	41594472	41594509	0.04	0.34763	0
chr13	43727819	43727984	0.17	-0.39427	0
chr13	45781178	45781603	0.43	0.29533	0.00064
chr13	47161167	47161752	0.59	0.37069	0
chr13	48986750	48987465	0.72	0.38209	0
chr13	50702410	50702675	0.27	0.31331	0
chr13	50702795	50703477	0.68	0.31058	0
chr13	50706810	50706830	0.02	0.30513	0.00021
chr13	100783450	100783618	0.17	0.39331	0
chr13	108234261	108234701	0.44	-0.29658	0.00042
chr13	111215148	111215290	0.14	-0.42531	0
chr13	111892737	111892794	0.06	0.32097	0
chr13	112236782	112236854	0.07	-0.30283	0.00021
chr13	112335939	112336027	0.09	-0.30447	0.00021
chr13	112554122	112554252	0.13	-0.31436	0
chr13	112555823	112555972	0.15	-0.31148	0
chr13	112624688	112624791	0.1	-0.28508	0.00042
chr13	112690122	112690517	0.4	-0.3056	0
chr13	112692119	112692270	0.15	-0.343	0
chr13	112759355	112759719	0.36	0.29076	0
chr13	112899567	112899619	0.05	-0.32632	0
chr13	112901907	112901929	0.02	-0.30658	0
chr13	112903693	112904449	0.76	-0.28508	0

chr13	112906400	112906711	0.31	-0.27538	0.00064
chr13	113556144	113556486	0.34	0.33712	0
chr13	113622038	113622102	0.06	-0.30034	0.00021
chr13	113648767	113648892	0.13	-0.38002	0
chr13	113649513	113649600	0.09	-0.29487	0.00064
chr13	113719136	113719172	0.04	-0.43732	0
chr13	113763037	113763122	0.09	-0.34811	0
chr13	113776873	113776964	0.09	0.34279	0
chr13	114056351	114056576	0.23	-0.29373	0.00064
chr13	114150007	114150139	0.13	0.27854	0.00042
chr14	21502873	21503144	0.27	0.30028	0.00021
chr14	23624778	23624788	0.01	0.32034	0
chr14	23706743	23706751	0.01	-0.31247	0
chr14	25597490	25597562	0.07	0.38366	0
chr14	32836157	32836446	0.29	0.31606	0
chr14	38727014	38727024	0.01	-0.30685	0
chr14	39703160	39703302	0.14	0.32228	0
chr14	45711848	45712025	0.18	0.46354	0
chr14	50559902	50560100	0.2	0.33906	0
chr14	50809390	50809588	0.2	0.33669	0
chr14	51290136	51290662	0.53	0.35392	0
chr14	52066950	52067152	0.2	0.41985	0
chr14	53256600	53256914	0.31	-0.35729	0
chr14	55153114	55153130	0.02	0.31899	0
chr14	55603874	55603957	0.08	0.36005	0
chr14	55707715	55707866	0.15	0.41087	0
chr14	57197411	57197764	0.35	0.30684	0
chr14	61108227	61108807	0.58	0.28024	0.00042
chr14	62009941	62010028	0.09	0.33387	0
chr14	64663864	64663928	0.06	0.34004	0
chr14	68978814	68979049	0.24	0.3083	0
chr14	68991156	68991371	0.22	0.35097	0
chr14	69052728	69052740	0.01	0.36947	0
chr14	69523223	69523229	0.01	0.36382	0
chr14	69754092	69754550	0.46	0.29539	0.00064
chr14	72053177	72053361	0.18	0.29509	0.00064
chr14	73146054	73146637	0.58	0.3706	0
chr14	73168082	73168188	0.11	0.29509	0.00064
chr14	73209128	73209188	0.06	0.31975	0
chr14	74257107	74257363	0.26	0.32917	0
chr14	74815085	74815131	0.05	0.33888	0
chr14	75704448	75704839	0.39	0.30972	0
chr14	76309091	76309157	0.07	0.37898	0
chr14	76445988	76446433	0.45	0.28255	0.00021
chr14	78021544	78021636	0.09	0.27509	0.00064
chr14	88502488	88502687	0.2	-0.29416	0
chr14	89817792	89818474	0.68	0.29412	0

chr14	94812770	94812826	0.06	-0.30254	0.00021
chr14	95078599	95078743	0.14	0.30058	0
chr14	96752011	96752231	0.22	0.31128	0
chr14	96967437	96967681	0.24	-0.32107	0
chr14	97262709	97262871	0.16	-0.34276	0
chr14	101155305	101155658	0.35	-0.2987	0
chr14	102027660	102027681	0.02	0.28619	0
chr14	103871225	103871452	0.23	0.40647	0
chr14	104896488	104897068	0.58	-0.31045	0
chr14	105512213	105512306	0.09	0.2947	0
chr14	106173575	106173722	0.15	-0.2925	0.00085
chr14	106198003	106198034	0.03	-0.29776	0.00021
chr15	31355304	31355362	0.06	0.31131	0
chr15	39458978	39459285	0.31	0.31139	0
chr15	40225341	40225618	0.28	-0.29621	0.00064
chr15	42749336	42749885	0.55	0.37267	0
chr15	42800679	42800833	0.15	0.36688	0
chr15	45005363	45005365	0	-0.32956	0
chr15	49715342	49715415	0.07	0.2928	0.00085
chr15	49716247	49716645	0.4	0.34893	0
chr15	52433022	52433433	0.41	0.31406	0
chr15	57029040	57029050	0.01	0.29362	0.00064
chr15	57510575	57510606	0.03	0.3146	0
chr15	59500970	59501408	0.44	0.29727	0
chr15	60285691	60285821	0.13	0.30959	0
chr15	60817947	60818455	0.51	0.27963	0.00042
chr15	61210082	61210235	0.15	0.32136	0
chr15	63358260	63358672	0.41	0.38204	0
chr15	64218300	64218534	0.23	0.30855	0
chr15	64795954	64796240	0.29	0.31768	0
chr15	65053605	65053614	0.01	0.29678	0.00042
chr15	70613427	70613797	0.37	0.30531	0
chr15	71389060	71389252	0.19	0.31143	0
chr15	74508376	74508523	0.15	-0.34275	0
chr15	77518882	77519170	0.29	0.31882	0
chr15	85330779	85330888	0.11	0.34431	0
chr15	85396292	85396513	0.22	0.44229	0
chr15	86215274	86215313	0.04	0.31817	0
chr15	86232932	86233220	0.29	0.3188	0
chr15	90388953	90389012	0.06	-0.32918	0
chr15	97345353	97345395	0.04	-0.30158	0.00021
chr15	101419275	101419333	0.06	-0.30456	0
chr16	1077597	1077624	0.03	-0.32819	0
chr16	1213672	1213919	0.25	-0.29366	0
chr16	1226269	1226302	0.03	-0.3047	0.00021
chr16	1245599	1245967	0.37	-0.30833	0
chr16	1271153	1272275	1.12	-0.30841	0

chr16	3149381	3149397	0.02	0.29385	0.00064
chr16	4289417	4289924	0.51	0.32698	0
chr16	5488787	5488843	0.06	-0.33425	0
chr16	5812000	5812252	0.25	-0.3024	0.00021
chr16	7949631	7949659	0.03	-0.30368	0.00021
chr16	11828669	11828819	0.15	0.40837	0
chr16	11886550	11886668	0.12	0.38502	0
chr16	12010646	12010867	0.22	-0.35007	0
chr16	14029456	14029472	0.02	0.39987	0
chr16	14280183	14280709	0.53	0.34546	0
chr16	15488936	15489190	0.25	-0.30791	0
chr16	16181015	16181129	0.11	0.32698	0
chr16	17609453	17609605	0.15	-0.33802	0
chr16	18939106	18939180	0.07	-0.33348	0
chr16	21012796	21012810	0.01	0.33054	0
chr16	22082727	22082857	0.13	0.36992	0
chr16	22296661	22296770	0.11	0.29658	0.00042
chr16	23153611	23153792	0.18	0.31215	0
chr16	27733016	27733133	0.12	0.33248	0
chr16	28224134	28224204	0.07	-0.3695	0
chr16	28635358	28635374	0.02	-0.35345	0
chr16	30456325	30456379	0.05	-0.3313	0
chr16	34741251	34741304	0.05	-0.33015	0
chr16	48277559	48277630	0.07	-0.3489	0
chr16	50403396	50403566	0.17	0.31198	0
chr16	67978429	67978450	0.02	0.303	0
chr16	69385547	69385827	0.28	0.30955	0
chr16	69832592	69832614	0.02	0.32089	0
chr16	69872614	69873116	0.5	0.29172	0
chr16	72232102	72232644	0.54	0.34033	0
chr16	76342377	76342620	0.24	-0.3174	0
chr16	78276006	78276299	0.29	0.30986	0
chr16	81271013	81271267	0.25	0.3	0.00021
chr16	82608833	82609548	0.72	-0.29679	0
chr16	83519231	83519418	0.19	-0.29273	0.00085
chr16	84828913	84829007	0.09	0.30751	0
chr16	85063708	85063787	0.08	-0.35495	0
chr16	88164417	88164581	0.16	-0.33632	0
chr16	88949367	88949579	0.21	-0.30761	0
chr17	579682	579774	0.09	0.36788	0
chr17	762510	763049	0.54	0.32366	0
chr17	800263	800431	0.17	0.34332	0
chr17	873255	873619	0.36	0.33916	0
chr17	1063892	1064017	0.13	-0.30153	0
chr17	2075719	2076468	0.75	0.33183	0
chr17	2140096	2140270	0.17	0.30313	0.00021
chr17	3377350	3377797	0.45	0.30273	0.00021

chr17	3716279	3716558	0.28	-0.30329	0
chr17	4108050	4108148	0.1	0.31706	0
chr17	4648682	4649076	0.39	0.30781	0
chr17	15522859	15522897	0.04	0.38251	0
chr17	16978535	16978673	0.14	0.30137	0.00021
chr17	17024192	17024437	0.25	0.29517	0.00064
chr17	18647507	18647524	0.02	0.36466	0
chr17	26500010	26500086	0.08	0.32811	0
chr17	30873196	30873447	0.25	0.36766	0
chr17	33823619	33823690	0.07	0.35222	0
chr17	33842255	33842301	0.05	0.32666	0
chr17	35423405	35423449	0.04	0.29254	0.00085
chr17	37216789	37216821	0.03	0.32674	0
chr17	38182487	38182512	0.03	0.29852	0.00021
chr17	46700424	46700449	0.03	0.29302	0.00085
chr17	46707338	46707781	0.44	0.32068	0
chr17	46709858	46710112	0.25	0.3077	0
chr17	48276255	48276347	0.09	0.29944	0.00021
chr17	57505491	57505597	0.11	0.3654	0
chr17	58216297	58216415	0.12	0.31551	0
chr17	60884617	60885154	0.54	-0.30905	0
chr17	61476730	61476846	0.12	0.34751	0
chr17	63070811	63071046	0.24	0.33936	0
chr17	64226823	64227024	0.2	0.32419	0
chr17	72620022	72620058	0.04	-0.32421	0
chr17	72666418	72666670	0.25	-0.31659	0
chr17	73128545	73128575	0.03	-0.31823	0
chr17	73955611	73955839	0.23	0.42589	0
chr17	74641167	74641294	0.13	0.28933	0
chr17	74868604	74868693	0.09	-0.27869	0.00042
chr17	75882353	75882609	0.26	-0.32148	0
chr17	76183632	76183641	0.01	-0.32298	0
chr17	76220608	76220955	0.35	0.34799	0
chr17	77997922	77997997	0.08	0.33927	0
chr17	79010831	79011140	0.31	-0.27176	0.00085
chr17	79380493	79380515	0.02	-0.30977	0
chr17	79381472	79381734	0.26	-0.32279	0
chr17	79388690	79388814	0.12	-0.2918	0.00085
chr17	79412681	79412788	0.11	-0.43859	0
chr17	79414215	79415034	0.82	-0.30325	0
chr17	79416203	79416550	0.35	-0.32541	0
chr17	79419796	79420145	0.35	-0.35596	0
chr17	79420279	79420436	0.16	-0.29113	0
chr17	79425877	79426049	0.17	-0.32917	0
chr17	79426353	79426432	0.08	-0.32359	0
chr17	79495664	79496290	0.63	-0.32357	0
chr17	80289437	80290106	0.67	-0.30644	0

chr17	80346849	80346912	0.06	-0.2963	0.00064
chr17	80627720	80627886	0.17	0.32829	0
chr17	80839243	80839375	0.13	0.35003	0
chr17	81006524	81006821	0.3	-0.30029	0.00021
chr18	3874746	3874883	0.14	0.30203	0.00021
chr18	5133855	5133933	0.08	-0.34986	0
chr18	5420951	5421105	0.15	-0.33973	0
chr18	10588980	10589360	0.38	-0.39059	0
chr18	20911287	20911360	0.07	-0.29931	0
chr18	33198928	33198945	0.02	0.38279	0
chr18	44775558	44775561	0	0.30355	0.00021
chr18	52627127	52627333	0.21	-0.32995	0
chr18	56296334	56296354	0.02	0.27317	0.00064
chr18	74199185	74199233	0.05	0.31888	0
chr18	75691297	75691425	0.13	-0.38336	0
chr18	75795498	75795691	0.19	-0.32493	0
chr18	75796593	75796626	0.03	-0.32763	0
chr18	76073399	76073557	0.16	-0.31392	0
chr18	76122391	76122611	0.22	-0.2946	0.00064
chr18	76260265	76260974	0.71	-0.31089	0
chr18	76304359	76304376	0.02	-0.31916	0
chr18	76400706	76401126	0.42	-0.37347	0
chr18	76445954	76446282	0.33	-0.33788	0
chr18	76477920	76478007	0.09	-0.29992	0.00021
chr18	76486314	76486611	0.3	-0.3202	0
chr18	76601647	76601755	0.11	-0.32899	0
chr18	76636062	76636159	0.1	-0.31776	0
chr18	76767645	76767684	0.04	-0.36048	0
chr19	1325931	1326119	0.19	-0.309	0
chr19	1851882	1851995	0.11	0.31019	0
chr19	2095466	2095643	0.18	-0.29353	0
chr19	3383483	3383726	0.24	0.30702	0
chr19	3917900	3917955	0.06	-0.32707	0
chr19	4555999	4556011	0.01	-0.33697	0
chr19	10928327	10928696	0.37	0.29779	0
chr19	11199222	11199476	0.25	0.29812	0.00021
chr19	11784647	11784774	0.13	0.28159	0
chr19	12624679	12624770	0.09	0.33546	0
chr19	13108155	13108217	0.06	0.30876	0
chr19	13113482	13113893	0.41	0.29695	0
chr19	13365923	13366101	0.18	-0.38503	0
chr19	15160160	15160318	0.16	-0.32411	0
chr19	32082614	32082879	0.27	-0.30981	0
chr19	34174388	34174777	0.39	-0.30619	0
chr19	36642720	36643070	0.35	0.31257	0
chr19	36643771	36643932	0.16	0.30998	0
chr19	38524517	38524950	0.43	-0.34851	0

chr19	50550284	50550492	0.21	0.3748	0
chr19	51898727	51898904	0.18	-0.32251	0
chr19	52452317	52452528	0.21	0.34859	0
chr19	54617570	54617654	0.08	-0.26532	0.00064
chr19	58220080	58220955	0.88	0.36748	0
chr19	58341492	58341619	0.13	0.32237	0
chr19	58715351	58716135	0.78	0.37221	0
chr19	59092612	59092692	0.08	0.34232	0
chr20	2308779	2308884	0.11	-0.34482	0
chr20	4906961	4906994	0.03	0.2998	0.00021
chr20	6015349	6015708	0.36	0.42462	0
chr20	7234895	7235067	0.17	-0.31373	0
chr20	11898478	11898557	0.08	0.30217	0
chr20	16555142	16555226	0.08	-0.34612	0
chr20	17557158	17557271	0.11	0.33235	0
chr20	17822792	17823154	0.36	0.32061	0
chr20	20180211	20180469	0.26	0.30103	0.00021
chr20	22541629	22541637	0.01	0.33238	0
chr20	22542761	22542854	0.09	0.31474	0
chr20	24929607	24929716	0.11	-0.33189	0
chr20	30639305	30639705	0.4	0.37577	0
chr20	33567656	33567887	0.23	0.3223	0
chr20	35924929	35925236	0.31	0.33094	0
chr20	37202230	37202274	0.04	0.32745	0
chr20	41597179	41597273	0.09	-0.29546	0
chr20	41691815	41691964	0.15	-0.31148	0
chr20	44116128	44116188	0.06	0.30355	0
chr20	46342767	46342769	0	0.31823	0
chr20	48551982	48551992	0.01	-0.32078	0
chr20	49737577	49738043	0.47	0.29648	0.00042
chr20	52198225	52198378	0.15	-0.30933	0
chr20	55199493	55200370	0.88	0.34211	0
chr20	56256274	56256437	0.16	-0.30609	0
chr20	56780599	56781279	0.68	-0.33009	0
chr20	58568926	58569157	0.23	0.32605	0
chr20	59087484	59087752	0.27	-0.32617	0
chr20	59654126	59654147	0.02	-0.37272	0
chr20	59835988	59836416	0.43	-0.31422	0
chr20	59866503	59866704	0.2	-0.35345	0
chr20	59968939	59969413	0.47	-0.38647	0
chr20	59987301	59987416	0.12	-0.30975	0
chr20	60033929	60034285	0.36	-0.35186	0
chr20	60074136	60074520	0.38	-0.3486	0
chr20	60119508	60119551	0.04	-0.47212	0
chr20	60242113	60242590	0.48	-0.30817	0
chr20	60254244	60254704	0.46	-0.3641	0
chr20	60303394	60303881	0.49	-0.30472	0.00021

chr20	60332183	60332244	0.06	-0.34242	0
chr20	60337213	60337350	0.14	-0.32255	0
chr20	60397766	60397915	0.15	-0.31126	0
chr20	60460170	60460568	0.4	-0.30293	0
chr20	60470081	60470267	0.19	-0.33202	0
chr20	62031739	62031924	0.19	-0.38553	0
chr20	62037718	62038206	0.49	-0.26165	0.00085
chr20	62052156	62052259	0.1	-0.30051	0.00021
chr20	62406677	62406721	0.04	0.36923	0
chr21	16374422	16374706	0.28	0.27162	0.00085
chr21	16774722	16774725	0	0.47249	0
chr21	32128785	32128800	0.02	-0.31539	0
chr21	32185331	32185365	0.03	-0.34564	0
chr21	34185960	34186122	0.16	0.28331	0.00021
chr21	38378189	38378406	0.22	0.31844	0
chr21	38821784	38821804	0.02	0.29545	0.00064
chr21	44161884	44162219	0.34	0.45631	0
chr21	44582429	44582492	0.06	-0.33985	0
chr21	45713704	45713719	0.02	-0.29571	0.00064
chr21	46850665	46850840	0.18	-0.29327	0
chr21	48081242	48081257	0.02	0.3176	0
chr22	18314772	18314866	0.09	0.31012	0
chr22	22901946	22902237	0.29	-0.35849	0
chr22	29977677	29977742	0.07	-0.34766	0
chr22	31836032	31836042	0.01	0.29663	0.00042
chr22	36278499	36278607	0.11	0.36227	0
chr22	37960396	37960426	0.03	0.3202	0
chr22	41904878	41905304	0.43	0.31648	0
chr22	48027277	48027487	0.21	-0.28435	0.00021
chr22	48027726	48027730	0	-0.29536	0.00064
chrX	2882183	2882333	0.15	0.30536	0
chrX	19082436	19082491	0.06	-0.30668	0
chrX	133680549	133680665	0.12	0.29896	0.00021
chrX	145077291	145077477	0.19	-0.29418	0
